# Synthesis and Pharmacological Evaluation of Novel Benzenesulfonamide Derivatives as Potential Anticonvulsant Agents

**DOI:** 10.3390/molecules200917585

**Published:** 2015-09-23

**Authors:** Zhiming Wang, Jinping Li, Xiao-Dong Zeng, Xian-Ming Hu, Xiaoju Zhou, Xuechuan Hong

**Affiliations:** State Key Laboratory of Virology, Key Laboratory of Combinatorial Biosynthesis and Drug Discovery, Ministry of Education, Wuhan University School of Pharmaceutical Sciences, 185 Donghu Road, Wuhan 430071, China; E-Mails: zhiming.wang@whu.edu.cn (Z.W.); lijinping19891210@126.com (J.L.); zxd201102@whu.edu.cn (X.-D.Z.); xmhu@whu.edu.cn (X.-M.H.)

**Keywords:** sulfonamide, anticonvulsant, MES, scPTZ

## Abstract

A novel series of benzenesulfonamide derivatives containing 4-aminobenzenesul-fonamide and α-amides branched valproic acid or 2,2-dimethylcyclopropanecarboxylic acid moieties were synthesized and screened for their anticonvulsant activities in mice maximal electroshock seizure (MES) and subcutaneous pentylenetetrazole (scPTZ) test. The activity experimental study showed that 2,2-dipropyl-*N*^1^-(4-sulfamoylphenyl)malonamide (**18b**) had the lowest median effective dose (ED_50_) of 16.36 mg/kg in MES test, and 2,2-dimethyl-*N*-(4-sulfamoylphenyl)cyclopropane-1,1-dicarboxamide (**12c**) had the lowest ED_50_ of 22.50 mg/kg in scPTZ test, which resulted in the protective indexe (PI) of 24.8 and 20.4, respectively. These promising data suggest the new compounds have good potential as new class of anticonvulsant agents with high effectiveness and low toxicity for the treatment of epilepsy.

## 1. Introduction

Epilepsy is a common neurological disorder that affects approximately 50 million people around the world [1,2,3]. In spite of the appearance of several novel antiepileptic drugs (AEDs) during the last two decades [4,5,6,7], it is still depressing that about 30% of epileptic patients fail to respond to the existing AEDs [8,9,10], which makes it necessary to focus on the development of active and safe AEDs accommodating wider range of people.

**Figure 1:**
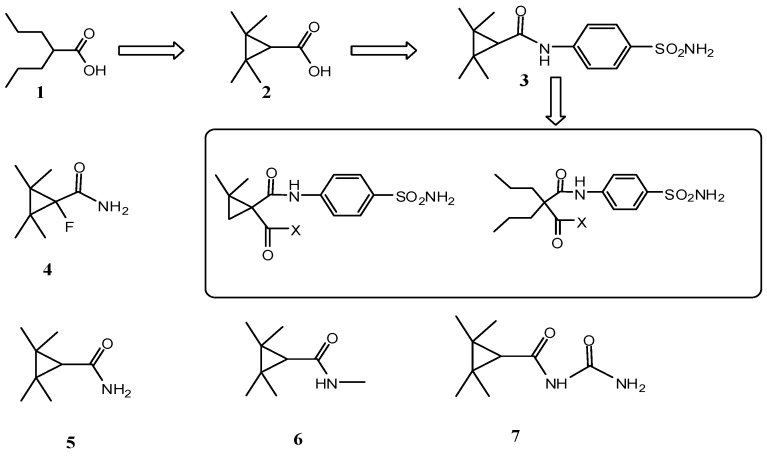
Structure of AEDs and designed compounds.

The clinical utilization of Valproic acid (VPA), one of the most widely used AEDs, is limited by its side effects such as teratogenicity and life-threatening hepatotoxicity [11,12,13]. The formation of hepatotoxic metabolites with a terminal double bond, 4-ene-VPA, is possibly the main cause of the hepatotoxicity [14,15]. In order to searching for new nonteratogenic and nonhepatotoxic compounds, numerous analogues and derivatives of VPA have been investigated. TMCA (**2**, Figure 1), a cyclic analogue of VPA, is found to be inactive at the rat anticonvulsant maximal electroshock seizure (MES) (ED_50_ > 150 mg/kg) model and can prevent its biotransformation to hepatotoxic metabolites [16,17]. Further studies have revealed that amide derivatives of **2**, TMCD (**5**), especially *N*-methoxy TMCD (MTMCD, **6**) and TMC-urea (**7**) are broad-spectrum anticonvulsants with a much wider safety margin than VPA [18]. In 2008, Jakob reported the synthesis and high potency of TMCD-benzenesulfonamide (**3**, ED_50_ = 26 mg/kg) along with a wide protective index (PI = TD_50_/ED_50_ >19) in the rat-MES test [19]. Recently, it was reported that α-fluoronated TMCD (**4**) was 120 times more potent than VPA in the rat-scMet test (ED_50_ = 6 mg/kg) with a remarkable protective index (PI = 20) [20]. These developments prompted us to focus on systematic structural modifications in the α position. In the current study, we introduced amid groups to the benzenesulfonamide system in order to get novel anticonvulsant agents. In view of the difficulty of synthesis, the 2,2,3,3-tetramethylcyclopropane fragment was replaced by 2,2-dimethylcyclopropane group, which has been proven to be a safe and effective structure in our group’s early work [21,22,23,24,25,26]. Herein, a series of benzenesulfonamide derivatives containing 4-amino-benzenesulfonamide and α-amides branched valproic acid or 2,2-dimethylcyclopropanecarboxylic acid moieties were synthesized and their pharmacological activities as potential anticonvulsant agents were evaluated in mice maximal MES and subcutaneous pentylenetetrazole (scPTZ) tests in this paper.

## 2. Results and Discussion

### 2.1. Chemistry

**Scheme 1:**
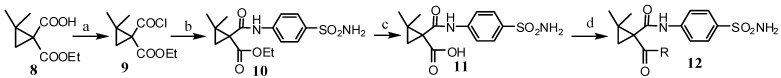
Synthetic route for the synthesis of compounds **12a**–**n**. Reagents and conditions: (**a**) SOCl_2_, DCM, refluxed, 2 h; (**b**) TEA, acetone, r.t, 4 h; (**c**) NaOH, EtOH, r.t, 12 h; and (**d**) EDCI, HOBt, DCM/THF, r.t, 12 h (r.t. means room temperature).

The general synthetic route for compound **12** is shown in Scheme 1. Compound **8**, used as the starting material, was reacted with SOCl_2_ in anhydrous DCM to obtain compound **9**. The coupling reaction of compound **9** and sulfonamide in the presenve of TEA resulted in the formation of compound **10** in 80% yield. Compound **11** was then obtained by hydrolysis of compound **10** in a 1 mol/L NaOH in EtOH–water (1:1) solution for 12 h. Amidation of compound **11** with a variety of amines in the EDCI/HOBt system gave the corresponding final products **12a**–**n** in good yields. The structures of all new compounds were characterized by ^1^H-NMR, ^13^C-NMR and MS. The spectrums were shown in supplementary materials.

**Scheme 2:**
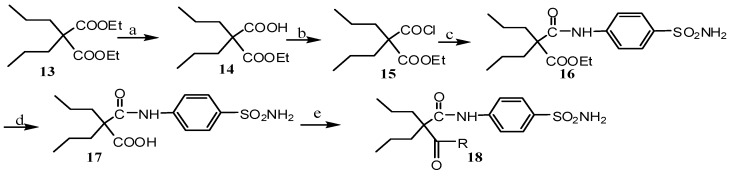
Synthetic route for the synthesis of compounds **18a**–**c**. Reagents and conditions: (**a**) NaOH, EtOH, r.t, 12 h; (**b**) SOCl2, DCM, refluxed, 2 h; (**c**) TEA, acetone, r.t, 4 h; (**d**) NaOH, EtOH, r.t, 12 h; and (**e**) EDCI, HOBt, DCM/THF, r.t, 12 h (r.t. means room temperature).

The synthetic route of compounds **18a**–**c** is listed in Scheme 2. It was very similar to that of compounds **12a**–**n**. However, all anilines and secondary amines failed to react with compound **17**. It may be attributed to the steric hindrance of the double propyl branch.

### 2.2. Pharmacological Evaluation

Chemical diversity and different biological mechanism of anticonvulsant drugs make it difficult to find a common method to identify the candidates. When it comes to develop novel anticonvulsant agents, it is necessary to apply conventional screening or structure modification of these tested compounds. Early identification of anticonvulsant agents is usually conducted via *in vivo* screening such as MES test and scPTZ test [27,28,29].

In the present study, 22 new synthesized compounds were evaluated following the standard procedure (NIH anticonvulsant drug development program). Those compounds were administrated intraperitoneally (i.p.) to male Kunming mice weighting 18–22 g. In the initial evaluation, the compounds were given at dose of 300 mg/kg, 100 mg/kg and 30 mg/kg. The observations were taken at two time intervals, namely 0.5 h and 4 h. The acute neurotoxicity was measured by the rotarod test. The results are summarized in Table 1.

**Table 1:** Anticonvulsant activity and neurotoxicity of compounds administered intraperitoneally.

Compounds		Intraperitioneal Injection into Mice ^a^						ClogP ^b^
		MES ^c^		scPTZ ^d^		Neurotoxocity ^e^		
No.	R	0.5 h	4 h	0.5 h	4 h	0.5 h	4 h	
**10**	-OEt	100	- ^f^	300	-	300	-	1.633
**11**	-OH	100	-	-	-	300	-	0.865
**12a**	-NHC(CH_3_)_3_	100	-	-	-	-	-	1.484
**12b**	-N(CH_2_CH_3_)_2_	100	300	-	-	300	-	1.735
**12c**	-NH_2_	30	100	30	100	300	-	−0.019
**12d**	-NHCH_3_	30	300	100	300	-	-	0.247
**12e**	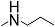	100	-	-	-	-	-	1.305
**12f**	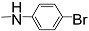	300	-	-	-	300	-	3.223
**12g**	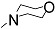	300	300	-	-	-	-	0.797
**12h**	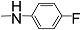	100	300	300	-	-	-	2.503
**12i**	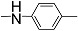	100	-	-	-	-	-	2.604
**12j**	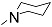	100	-	-	-	-	-	1.591
**12k**	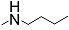	100	-	300	-	300	-	1.834
**12l**	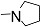	-	-	-	-	-	-	1.032
**12m**	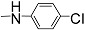	300	-	-	-	-	-	3.071
**12n**	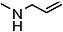	100	300	-	-	-	-	1.021
**16**	-OEt	300	-	-	-	300	-	2.815
**17**	-OH	100	-	300	-	300	-	2.046
**18a**	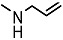	100	300	-	-	-	-	2.203
**18b**	-NH_2_	30	30	-	-	300	-	1.163
**18c**	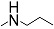	100	100	-	-	-	-	2.487
Phenytoin ^g^		30	30	-	-	100	100	
Ethosuximide ^h^		-	-	100	300	-	-	

^a^: 30 mg/kg, 100 mg/kg, and 300 mg/kg of doses were administered i.p. The data in the table indicate the minimal dose whereby bioactivity was demonstrated. The animals were examined at 0.5 h and 4.0 h after injection was administered; ^b^: Clog P was calculated using software ChemDraw Ultra (version 6.0.1; PerkinElmer Informatics, Waltham, MA, USA); ^c^: Maximal electroshock test; ^d^: Subcutaneous pentylenetetrazole test; ^e^: Neurotoxocity screening; ^f^: A dash indicates the absence of anticonvulsant activity and neurotoxicity at the maximum dose administered (300 mg/kg); ^g^: Data from Reference [30]; ^h^: Data from Reference [31].

The initial evaluation of all these compounds, with the exception of **12l**, showed anticonvulsant activities in the mice i.p. MES screening. Among 2,2-dimethyl-*N*-(4-sulfamoylphenyl)cyclopropane-1,1-dicarboxamide derivatives (**12a**–**12n**), the most active analogs were **12c** and **12d**, which showed anticonvulsant activities at 0.5 h after administrated intraperitoneally (i.p.) at a dose of 30 mg/kg to the male Kunming mice. Furthermore, compound **12c** was active at 4 h at a dose of 100 mg/kg and had a longer duration, while compound **12d** did not show any improvements. The compounds containing aromatic amines substituents such as **12h** and **12i** acted much better than **12f** and **12n**, which indicated that substituents with the phenyl moiety had significant influence on the anticonvulsant activity. When the substituents at the carboxyl position were alkyl amines, the order of anticonvulsant activity against the MES test was found to be **12d** > **12e** = **12n** > **12k** = **12a**. It is noteworthy that the anticonvulsant activity of those compounds was decreased when the alkyl moiety possesses more carbons. A double bond was introduced to the branch in compounds **12n** and **18a** in order to obtain a short-duration anticonvulsant agent like secobarbital, which also contained a carbon–carbon double bond within the molecular structure [32]. Unexpectedly, both compounds **12n** and **18a** showed the anticonvulsant activity at 4 h after administrated with a dose of 300 mg/kg. Speaking of the performance of compounds **12c** and **18b**, both containing the same amino-group, compound **18b** showed anticonvulsant activity at 4 h after administrated with a dose of 30 mg/kg, while compound **12c** showed anticonvulsant activity with a dose of 100 mg/kg, which may be attributed to the lipophilicity. Meanwhile, when compounds **18c** and **12e** were compared, a similar activity was obtained.

In the scPTZ test, only six compounds exhibited protection against induced seizure at 300 mg/kg or less. Compounds **10**, **12h**, **12k** and **17** showed anticonvulsant activity at 300 mg/kg after 0.5 h, while compound **12c** was more active at 30 mg/kg after 0.5 h. Compound **12c** was still active after 4 h at a dose of 100 mg/kg. The result indicated that compound **12c** had a rather quick onset and was more active than the positive control drug ethosuximide in the scPTZ screen.

All the tested compounds maintained balance well in the neurotoxicity test at 100 mg/kg or less. However, compounds **10**, **11**, **12b**, **12c**, **12f**, **12k**, **16**, **17** and **18b** revealed neurotoxicity at a dose of 300 mg/kg after 0.5 h and turned out to be non-toxic after 4 h. All the most active molecules, namely **12c**, **12d** and **18b**, showed anticonvulsant potencies in lower doses than neurotoxic properties. No relation between the activity and the neurotoxocity was found. In other words, better activity did not mean more neurotoxocity.

Crossing the blood–brain barrier (BBB) is an important factor influencing anticonvulsant activity. It is believed that ClogP (calculated LogP) values between 1 and 2 is sufficient for crossing BBB and the lipophilicity of the titled compound is very important in the central nervous system drug penetrating through BBB [33]. The ClogP (calculated LogP) values of the tested compounds were also listed in Table 1. The above data did not show an obvious correlation between ClogP values and *in vivo* anticonvulsant activities. The most activity compounds, **12c** and **12d**, showed relatively lower ClogP of −0.019 and 0.247. These values suggested a low concentration of those compounds in brain.

**Figure 2:**
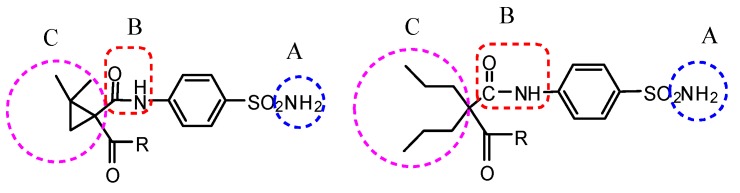
Pharmacophoric structure of designed compounds.

There are two possible reasons for the existence of anticonvulsant activity. The designed compounds meet a general structural model of AEDs as shown in Figure 2 [34]. At first, the dimethylcyclopropane group or the double propyl branch can act as hydrophobic group to enable the compounds to penetrate through the BBB. At the same time, the amide groups act as hydrogen binding domain, while amino as an electron donor group. On the other hand, these compounds may act as carbonic anhydrase inhibitor.

In view of the excellent performance of compounds **12c**, **12d** and **18b** in the initial screening, they were selected for Phase-II screening. The resulting results for these three molecules were shown in Table 2. Approximated time of peak effects (TPE) of compounds **12c**, **12d** and **18b** were 1 h, 1 h and 2 h, respectively. The quantitative evaluation of pharmacological parameters (ED_50_ and TD_50_) was performed at TPE after administrated intraperitoneally. Compound **18b**, the most active compound, showed an ED_50_ value of 16.36 mg/kg among the tested compounds in the MES screening. Compound **12d** exhibited the lowest toxicity with a TD_50_ value >500 mg/kg. Compound **12c**, which turned out to be seven times more active than that of valproate, revealed the best activity in the scPTZ test with an ED_50_ value of 22.50 mg/kg. All these three compounds mentioned above possessed higher PI than that of standard drugs (e.g., Phenytoin and valproate). Consequently, the higher anticonvulsant activity of compound **18b** (PI = 24.8) in the MES test and compound **12c** (PI = 20.4) in scPTZ test as compared to VPA and its substantially wider safety margin than VPA and Phenytoin were observed. These observations indicated this benzenesulfonamide derivative containing 4-aminobenzenesulfonamide and the α-amide branched valproic acid or 2,2-dimethylcyclopropanecarboxylic acid moiety was a potential candidate as a new AED.

**Table 2:** Quantitative anticonvulsant data in mice dosed intraperitoneally.

Compound	ED_50_^a^		TD_50_^b^	PI ^c^	
	MES	scPTZ		MES	scPTZ
12c	24.47 (20.05–29.83) ^d^	22.50 (16.25–31.14)	499.2 (455.3–547.4)	20.4	22.19
12d	25.25 (18.14–35.14)	ND ^e^	>500	>19.80	ND ^e^
18b	16.36 (14.17–18.89)	ND ^e^	406.7 (337.7–489.7)	24.85	ND ^e^
phenytoin ^f^	9.5 (8.1–10.4)	>300	65.5	6.9	<0.22
valproate ^f^	272	149	426	1.6	2.9

^a^: Dose in milligrams per kilogram body mass; ^b^: Minimal toxicity which was determined by rotarod test 30 min after the test drug was administered; ^c^: Protection index (TD_50_/ED_50_); ^d^: Data in parentheses are the 95% confidence limits; ^e^: Not determined; ^f^: Data from Reference [33].

The acute toxicity of compounds **12c**, **12d** and **18b** were measured and the results were reported in Table 3 and Table 4. In the preliminary test, all compounds dosed intragastric administration did not show acute toxicity to kill mice at 2000 mg/kg. Unfortunately, when these compounds were administrated intraperitoneally, some mice died at 500 mg/kg or 2000 mg/kg. The LD_50_ values of the tested agents administered intraperitoneally, along with the data on the standard drug (phenytoin), are listed in Table 4. Compound **12c** showed a LD_50_ of 762.7 mg/kg; compound **12d**, the least toxic compound, showed a LD_50_ of 922.0 mg/kg, while the LD_50_ value of phenytoin was 100 mg/kg. These results proved that the newly synthesized compounds were safer than the standard drug. When comparing **12c** with **18b**, it was self-evident that lower LD_50_ came with lower ED_50_.

**Table 3:** The acute toxicity of compounds in preliminary test.

Compound	Dose ^a^	Administration Route	
		i.g ^b^	i.p ^c^
**12c**	2000	0/5 ^d^	4/5
	500	ND ^e^	1/5
	50	ND	0/5
**12d**	2000	0/5	5/5
	500	ND	1/5
	50	ND	0/5
**18b**	2000	0/5	5/5
	500	ND	2/5
	50	ND	0/5

^a^: Dose in milligrams per kilogram body mass; ^b^: Intragastric injection; ^c^: Intraperitoneal injection; ^d^: Number of animals protected/number of animals tested; ^e^: Not determined.

**Table 4:** The acute toxicity of compounds in intrapertioneal injection.

Compound	LD_50_^a^
**12c**	762.7 (656.8–885.6)
**12d**	922.0 (601.1–1414)
**18b**	638.0 (475.0–857.0)
**phenytoin**	100 (94.3–101.2)

^a^: Dose in milligrams per kilogram body mass.

## 3. Experimental Section

### 3.1. General Information

All commercially available solvents and reagents were purchased from Sigma-Aldrich (St. Louis, MO, USA) and used without further purification. ^1^H- and ^13^C-NMR spectra were recorded on Bruker AV400 MHz spectrometers (Bruker Biospin, Rheinstetten, Germany) in deuteriochloroform (CDCl_3_) unless otherwise stated. All ^1^H chemical shifts are reported in ppm (δ) relative to TMS (0.00); ^13^C shifts are reported in ppm (δ) relative to CDCl_3_ (77.0). Data are reported in the following order: chemical shifts are given (δ); multiplicities are indicated as br (broadened), s (singlet), d (doublet), t (triplet), q (quartet), m (multiplet), app (apparent); coupling constants, *J*, are reported (Hz); integration is provided. Uncalibrated melting points were taken on XT-4 apparatus (Yuhua Instruments Co., Ltd., Gongyi, Henan, China). Analytical thin-layer chromatography (TLC) was performed on Merck silica gel aluminum sheets (Merck KGaA, Darmstadt, Germany) with F-254 indicator. Visualization was accomplished by UV light, or with solutions of K_2_CO_3_/KMnO_4_ in water. Purification by chromatography was performed using 200–300 mesh SiO_2_ (Qingdao Haiyang Chemical Co., Ltd., Qingdao, Shandong, China) with compressed air as a source of positive pressure. The mass spectra were obtained on Applied Biosystem/MDS-SCIEX API 2000 (AB Sciex Pte. Ltd., Framingham, MA, USA) with Agilent HPLCs, and MicrOTOF-Q orthogonal-accelerated TOF mass spectrometer (Bruker Daltonics, Bremen, Gremany) with an ESI source. Solvents for reactions and chromatography were reagent grade and used as received. “Brine” refers to a saturated aqueous solution of NaCl.

### 3.2. Synthesis

#### 3.2.1. Ethyl 1-(Chlorocarbonyl)-2,2dimethylcyclopropanecarboxylate **10**

Compound **8** (1.86 g) was dissolved in SO_2_Cl_2_ (10 mL) before the mixture was heated to reflux for 2 h. Reaction completion was monitored by TLC analysis. Then, this mixture was cooled to room temperature and concentrated. The resulting oil was dissolved in dry acetone (10 mL) and was transferred dropwise to a solution of sulfonamide (1.7 g ) in dry acetone (20 mL) in the presence of 2 Et_3_N (mL) and catalytic amount of DMAP. The mixture was completed in 4 h at room temperature. Then, the reaction mixture was diluted with ethyl acetate (30 mL), the organic layer was washed with 1 mol/L HCl (10 mL × 2), water (10 mL × 2) and brine (10 mL) subsequently. The organic phase was dried over MgSO_4_ and concentrated under vacuum. The obtained crude product was purified by column chromatography (EtOAc:Petroleum ether = 1:1) to give the titled compound **10** as a white solid. Yield: 90%; mp: 138–140 °C; ^1^H-NMR (400 MHz, DMSO) δ 10.40 (s, -CONH, 1H), 7.86–7.66 (m, 4H, ArH), 7.25 (s, 2H, SO_2_NH_2_), 4.21–4.00 (m, 2H, -CH2CH3), 1.38 (d, *J* = 4.8 Hz, 1H, Cpr-CH), 1.37 (d, *J* = 4.8 Hz, 1H, Cpr-CH), 1.33 (s, 3H, Me), 1.18 (t, *J* = 7.1 Hz, 3H, -CH2CH3), 1.12 (s, 3H, Me). ^13^C-NMR (101 MHz, MeOD) δ 171.4, 168.3, 143.4, 139.8, 128.2, 120.67, 62.8, 42.8, 31.7, 26.5, 23.3, 20.4, 14.5. HRMS-ESI Calcd. for C_15_H_21_N_2_O_5_S [M + H]^+^: 341.1171; Found: 341.1433.

#### 3.2.2. 2,2-Dimethyl-1-(4-sulfamoylphenylcarbamoyl)cyclopropanecarboxylic Acid **11**

1 mol/L sodium hydroxide (12 mL) was added dropwise to a solution of compound **10** (3.4 g) in ethanol (20 mL). The resulting mixture was stirred at room temperature for 18 h. EtOH was removed under reduced pressure and the mixture was extracted with ethyl acetate (10 mL × 2). The remaining aqueous layer was acidified to pH 2 by 3 mol/L HCl, extracted with ethyl acetate (10 mL × 2). The organic layer was concentrated to give a pale yellow oil and further crystallized by ethyl ether to give compound **11** as a white solid. Yield: 88%; mp: 180–183 °C; ^1^H-NMR (400 MHz, DMSO) δ 12.87 (s, 1H), 10.41 (s, 1H), 7.95–7.62 (m, 4H), 7.24 (s, 2H), 1.35 (d, *J* = 4.5 Hz, 1H), 1.33 (s, 3H), 1.31 (d, *J* = 4.5 Hz, 1H), 1.10 (s, 3H).^13^C-NMR (101 MHz, MeOD) δ 173.3, 168.8, 143.4, 139.7, 128.2, 120.6, 42.5, 31.7, 26.3, 23.0, 20.6. HRMS-ESI Calcd. for C_13_H_16_N_2_NaO_5_S [M + Na]^+^: 335.0672; Found: 335.0826.

#### 3.2.3. General Produce for the Synthesis of Compounds **12a**–**n**

Compound **11** (0.62 g) and HOBt (0.27 g) were dissolved in anhydrous tetrahydrofuran (10 mL) and was transferred to a stirred solution of EDCI (0.4 g) in dicolormethane (10 mL). After 0.5 h, a suitable amine (2 mmol) in tetrahydrofuran (5 mL) was added, the reaction was monitored by TLC analysis and completed in 12–24 h. The solvents were evaporated under vacuum to give a yellow oil, which was chromatographed (Petroleum ether–EtOAc system) to give compounds **12a**–**n** as white solids.

*N-tert-Butyl-2,2-dimethyl-N-(4-sulfamoylphenyl)cyclopropane-1,1-dicarboxamide* (**12a**): Yield: 70%; mp: 212–215 °C; ^1^H-NMR (400 MHz, DMSO) δ 10.33 (s, 1H, CONH), 7.76 (s, 4H, ArH), 7.59 (s, 1H, NH), 7.27 (s, 2H, SO_2_NH_2_), 1.47 (d, *J* = 5.5 Hz, 1H, Cpr-CH), 1.40 (d, *J* = 5.3 Hz, 1H, Cpr-CH), 1.27 (s, 9H, Me), 1.10 (s, 3H, Me), 1.06 (s, 3H, Me ).^13^C-NMR (101 MHz, MeOD) δ 169.8, 169.7, 142.8, 140.2, 128.2, 120.8, 52.7, 44.6, 28.8, 28.0, 23.3, 21.8, 21.6. MS-ESI [M + H]^+^ 368.2. HRMS-ESI Calcd. for C_17_H_25_N_3_NaO_4_S [M + Na]^+^: 390.1463; Found: 390.1567.

*N,N-Diethyl-2,2-dimethyl-N-(4-sulfamoylphenyl)cyclopropane-1,1-dicarboxamide* (**12b**): Yield: 75%; mp: 156–158 °C; ^1^H-NMR (400 MHz, DMSO) δ 9.83 (s, 1H, CONH), 7.79 (d, *J* = 8.9 Hz, 2H, ArH), 7.73 (d, *J* = 8.9 Hz, 2H, ArH), 7.25 (s, 2H, SO_2_NH_2_), 3.32 (m, 4H, -CH2CH3), 1.37 (d, *J* = 4.9 Hz, 1H, Cpr-CH), 1.16 (s, 3H, Me), 1.14 (d, *J* = 5.0 Hz, 1H, Cpr-CH), 1.10 (s, 3H, Me), 1.05 (t, *J* = 6.9 Hz, 3H, Me), 1.00 (t, *J* = 7.0 Hz, 3H, Me).^13^C-NMR (101 MHz, MeOD) δ 170.5, 168.1, 143.0, 140.1, 128.4, 128.3, 120.6, 43.8, 43.1, 41.2, 28.0, 25.4, 24.1, 21.5, 14.1, 12.8. MS-ESI [M + H]^+^: 368.2. HRMS-ESI Calcd. for C_17_H_25_N_3_NaO_4_S [M + Na]^+^: 390.1463; Found: 390.1561.

*2,2-Dimethyl-N-(4-sulfamoylphenyl)cyclopropane-1,1-dicarboxamide* (**12c**): Yield: 60%; mp: 197–200 °C; ^1^H-NMR (400 MHz, DMSO) δ 10.60 (s, 1H, CONH), 7.76 (s, 4H, ArH), 7.54 (d, *J* = 5.2 Hz, 2H, NH_2_), 7.26 (s, 2H, SO_2_NH_2_), 1.46 (d, *J* = 5.5 Hz, 1H, Cpr-CH), 1.44 (d, *J* = 5.5 Hz, 1H, Cpr-CH), 1.15 (s, 3H, Me), 1.08 (s, 3H, Me).^13^C-NMR (101 MHz, MeOD) δ 173.7, 169.0, 142.9, 140.0, 128.3, 120.8, 43.6, 28.7, 24.3, 23.4, 21.7, 21.6. MS-ESI [M + H]^+^: 312.1. HRMS-ESI Calcd. for C_13_H_17_N_3_NaO_4_S [M + Na]^+^: 334.0837; Found: 334.1000.

*N,2,2-Trimethyl-N-(4-sulfamoylphenyl)cyclopropane-1,1-dicarboxamide* (**12d**): Yield: 80%; mp: 217–220 °C; ^1^H-NMR (400 MHz, DMSO) δ 10.56 (s, 1H, CONH), 8.02 (d, *J* = 4.6 Hz, 1H, NH), 7.76 (s, 4H, ArH), 7.27 (s, 2H, SO_2_NH_2_), 2.65 (d, *J* = 4.6 Hz, 3H, Me), 1.44 (s, 2H, Cpr-CH), 1.08 (s, 6H, Me). ^13^C-NMR (101 MHz, MeOD) δ 171.8, 168.6, 142.9, 134.0, 128.3, 120.6, 43.9, 28.6, 26.7, 22.9, 21.9, 21.3. MS-ESI [M + H]^+^: 326.1. HRMS-ESI Calcd. for C_14_H_19_N_3_NaO_4_S [M + Na]^+^: 348.0994; Found: 348.1202.

*2,2-Dimethyl-N-propyl-N-(4-sulfamoylphenyl)cyclopropane-1,1-dicarboxamide* (**12e**): Yield: 70%; mp: 180–183 °C; ^1^H-NMR (400 MHz, DMSO) δ 10.53 (s, 1H, CONH), 8.09 (t, *J* = 5.7 Hz, 1H, NH), 7.76 (s, 4H, ArH), 7.27 (s, 2H, SO_2_NH_2_), 3.19–2.95 (m, 2H, CH_2_), 1.47(d, *J* = 7.2 Hz, 1H, Cpr-CH), 1.45 (m, 2H, CH_2_), 1.42 (d, *J* = 7.2 Hz, 1H, Cpr-CH), 1.09 (s, 3H, Me), 1.08 (s, 3H, Me), 0.83 (t, *J* = 7.4 Hz, 3H, Me).^13^C-NMR (101 MHz, MeOD) δ 176.0, 174.2, 142.8, 140.1, 128.2, 121.4, 59.0, 42.5, 40.9, 23.5, 19.5, 14.6, 11.8. MS-ESI [M + H]^+^: 354.1. HRMS-ESI Calcd. for C_16_H_23_N_3_NaO_4_S [M + Na]^+^ : 376.1307; Found: 376.1458.

*N-(4-Bromophenyl)-2,2-dimethyl-N-(4-sulfamoylpheny-l)cyclopropane-1,1-dicarboxamide* (**12f**): Yield: 50%; mp: 199–201 °C; ^1^H-NMR (400 MHz, DMSO) δ 10.24 (s, 1H, CONH), 10.05 (s, 1H, NH), 7.78 (q, *J* = 9.1 Hz, 4H, ArH), 7.61 (d, *J* = 8.9 Hz, 2H, ArH), 7.52 (t, *J* = 8.7 Hz, 2H, ArH), 7.28 (s, 2H, SO_2_NH_2_), 1.59 (s, 2H, Cpr-CH), 1.17 (s, 6H, Me). ^13^C-NMR (101 MHz, DMSO) δ 166.7, 141.1, 139.0, 137.5, 131.5, 126.6, 122.2, 119.6, 115.7, 43.4, 27.0, 22.8, 21.4, 21.2. MS-ESI [M + H]^+^: 467.0. HRMS-ESI Calcd. for C_19_H_20_BrN_3_NaO_4_S [M + Na]^+^: 488.0256, 490.0235; Found: 488.0389, 490.0376.

*2,2-Dimethyl-1-(morpholine-4-carbonyl)-N-(4-sulfamoylphenyl)cyclopropanecarboxamide* (**12g**): Yield: 65%; mp: 170–173 °C;^1^H-NMR (400 MHz, DMSO) δ 9.93 (s, 1H, CONH), 7.78 (m, 4H, ArH), 7.27 (s, 2H, SO_2_NH_2_), 3.53 (m, 8H, CH_2_), 1.39 (d, *J* = 5.1 Hz, 1H, Cpr-CH), 1.16 (s, 3H, Me), 1.14 (d, *J* = 5.1 Hz, 1H, Cpr-CH), 1.12 (s, 3H, Me).^13^C-NMR (101 MHz, MeOD) δ 169.6, 168.1, 143.0, 140.2, 128.2, 127.1, 120.8, 66.8, 43.0, 27.9, 25.4, 23.9, 21.7, 15.5. MS-ESI [M + H]^+^: 382.1. HRMS-ESI Calcd. for C_17_H_23_N_3_NaO_5_S [M + Na]^+^: 404.1256; Found: 404.1285.

*N-(4-Fluorophenyl)-2,2-dimethyl-N-(4-sulfamoylphenyl)cyclopropane-1,1-dicarboxamide* (**12h**): Yield: 60%; mp: 200–203 °C;^1^H-NMR (400 MHz, DMSO) δ 10.27 (s, 1H, CONH), 9.99 (s, 1H, NH), 7.88–7.70 (m, 4H, ArH), 7.70–7.55 (m, 2H, ArH), 7.28 (s, 2H, SO_2_NH_2_), 7.17 (dd, *J* = 12.3, 5.5 Hz, 2H, ArH), 1.59 (s, 2H, Cpr-CH), 1.17 (s, 3H, Me), 1.15 (s, 3H, Me).^13^C-NMR (101 MHz, MeOD) δ 169.2, 169.0, 162.3, 159.9, 142.7, 140.2, 135.2, 128.2, 124.0, 124.0, 121.0, 116.5, 116.3, 44.7, 29.2, 23.5, 22.0, 21.6. MS-ESI [M + H]^+^: 406.1. HRMS-ESI Calcd. for C_17_H_23_N_3_NaO_5_S [M + Na]^+^: 404.1256; Found: 404.1285.

*2,2-Dimethyl-N-(4-sulfamoylphenyl)-N-p-tolylcyclopropane-1,1-dicarboxamide* (**12i**): Yield: 70%; mp: 134–136 °C;^1^H-NMR (400 MHz, DMSO) δ 10.31 (s, 1H, CONH), 9.85 (s, 1H, NH), 7.78 (q, *J* = 9.0 Hz, 4H, ArH), 7.49 (d, *J* = 8.3 Hz, 2H, ArH), 7.27 (s, 2H, SO_2_NH_2_), 7.13 (d, *J* = 8.2 Hz, 2H, ArH), 2.26 (s, 3H, Me), 1.60 (d, *J* = 5.7 Hz, 1H, Cpr-CH), 1.58 (d, *J* = 5.7 Hz, 1H, Cpr-CH), 1.16 (s, 3H, Me), 1.14 (s, 3H, Me).^13^C-NMR (101 MHz, MeOD) δ 169.1, 169.0, 142.7, 140.2, 137.9, 130.7, 129.9, 128.2, 123.2, 121.0, 44.8, 29.3, 23.6, 21.9, 21.7. MS-ESI [M + H]^+^: 402.1. HRMS-ESI Calcd. for C_20_H_23_N_3_NaO_4_S [M + Na]^+^: 424.1307; Found: 424.1422.

*2,2-Dimethyl-1-(piperidine-1-carbonyl)-N-(4-sulfamoylpenyl)cyclopropanecarboxamide* (**12j**): Yield: 50%; mp: 207–209 °C; ^1^H-NMR (400 MHz, DMSO) δ 9.79 (s, 1H, CONH), 7.76 (d, 8.9 Hz, 2H, ArH), 7.56 (d, 8.9 Hz, 2H, ArH), 7.25 (s, 2H, SO_2_NH_2_), 3.75 (s, 1H, CH_2_), 3.51 (s, 2H, CH_2_), 3.38 (s, 1H, CH_2_), 1.44 (m, 6H, CH_2_), 1.40 (d, *J* = 4.9 Hz, 1H, Cpr-CH), 1.15 (s, 3H, Me), 1.11 (s, 3H, Me), 1.09 (d, *J* = 5.0 Hz, 1H, Cpr-CH).^13^C-NMR (101 MHz, MeOD) δ 169.5, 168.1, 143.0, 140.1, 128.2, 120.6, 43.0, 30.7, 28.2, 27.6, 26.8, 25.6, 25.3, 24.0, 21.5. MS-ESI [M + H]^+^: 380.1. HRMS-ESI Calcd. for C_18_H_25_N_3_NaO_4_S [M + Na]^+^: 402.1463; Found: 402.1567.

*N-Butyl-2,2-dimethyl-N-(4-sulfamoylphenyl)cyclopropane-1,1-dicarboxamide* (**12k**): Yield: 57%; mp: 170–172 °C; ^1^H-NMR (400 MHz, DMSO) δ 10.53 (s, 1H, CONH), 8.08 (t, *J* = 5.6 Hz, 1H, NH), 7.75 (s, 4H, ArH), 7.26 (s, 2H, SO_2_NH_2_), 3.25– 2.98 (m, 2H, CH_2_), 1.45 (s, 2H, Cpr-CH), 1.40 (m, 2H, CH_2_), 1.26 (m, 2H, CH_2_), 1.09 (s, 3H, Me), 1.08 (s, 3H, Me), 0.86 (t, *J* = 7.3 Hz, 3H, Me).^13^C-NMR (101 MHz, MeOD) δ 171.1, 168.8, 142.8, 140.0, 128.3, 120.6, 44.0, 40.7, 32.5, 28.4, 23.0, 22.0, 21.4, 21.2, 14.1. MS-ESI [M + H]^+^: 368.1. HRMS-ESI Calcd. for C_17_H_25_N_3_NaO_4_S [M + Na]^+^: 390.1463; Found: 390.1566.

*2,2-Dimethyl-1-(pyrrolidine-1-carbonyl)-N-(4-sulfamoylphenyl)cyclopropanecarboxamide* (**12l**): Yield: 39%; mp: 225–227 °C; ^1^H-NMR (400 MHz, DMSO) δ 9.88 (s, 1H, CONH), 7.76 (q, *J* = 9.0 Hz, 4H, ArH), 7.27 (s, 2H, SO_2_NH_2_), 3.53 (m, 2H, CH_2_), 3.35 (m, 2H, CH_2_), 1.92–1.78 (m, 2H, CH_2_), 1.78–1.64 (m, 2H, CH_2_), 1.39 (d, *J* = 5.0 Hz, 1H, Cpr-CH), 1.22 (d, *J* = 5.0 Hz, 1H, Cpr-CH), 1.15 (s, 3H, Me), 1.12 (s, 3H, Me). MS-ESI [M + H]^+^: 366.1. HRMS-ESI Calcd. for C_17_H_23_N_3_NaO_4_S [M + Na]^+^: 388.1307; Found: 388.1356.

*N-(4-Chlorophenyl)-2,2-dimethyl-N-(4-sulfamoylphenyl)cyclopropane-1,1-dicarboxamide* (**12m**): Yield: 70%; mp: 210–213 °C;^1^H-NMR (400 MHz, DMSO) δ 10.26 (s, 1H, CONH), 10.07 (s, 1H, NH), 7.78 (m, 4H, ArH), 7.67 (d, *J* = 8.8 Hz, 2H, ArH), 7.38 (d, *J* = 8.8 Hz, 2H, ArH), 7.28 (s, 2H, SO_2_NH_2_), 1.59 (s, 2H, Cpr-CH), 1.16 (d, *J* = 3.0 Hz, 6H, Me). ^13^C-NMR (101 MHz, MeOD) δ 169.1, 169.0, 142.7, 140.2, 137.9, 130.7, 129.9, 128.2, 123.2, 121.0, 44.8, 29.3, 23.6, 21.9, 21.7. MS-ESI [M + H]^+^: 422.0. HRMS-ESI Calcd. for C_19_H_20_ClN_3_NaO_4_S [M + Na]^+^: 444.0761; Found: 444.0884.

*N-Allyl-2,2-dimethyl-N-(4-sulfamoylphenyl)cyclopropane-1,1-dicarboxamide* (**12n**): Yield: 80%; mp: 179–181 °C; ^1^H-NMR (400 MHz, DMSO) δ 10.46 (s, 1H, CONH), 8.23 (t, *J* = 5.7 Hz, 1H, NH), 7.76 (s, 4H, ArH), 7.27 (s, 2H, SO_2_NH_2_), 5.92– 5.67 (m, 1H, CH), 5.29– 4.96 (m, 3H, Me), 3.77 (m, 3H, Me), 1.49 (d, *J* = 5.5 Hz, 1H, Cpr-CH), 1.45 (d, *J* = 5.5 Hz, 1H, Cpr-CH), 1.10 (s, 3H, Me), 1.09 (s, 3H, Me). ^13^C-NMR (101 MHz, MeOD) δ 170.9, 168.8, 142.8, 140.0, 135.2, 128.3, 120.7, 116.6, 44.0, 43.2, 28.5, 23.1, 21.9, 21.5. MS-ESI [M + H]^+^: 352.1. HRMS-ESI Calcd. for C_16_H_21_N_3_NaO_4_S [M + Na]^+^: 374.1150; Found: 374.1182.

#### 3.2.4. Ethyl 2-Propyl-2-(4-sulfamoylphenylcarbamoyl)pentanoate **16**

*Ethyl 2-propyl-2-(4-sulfamoylphenylcarbamoyl)pentanoate* (**16**): Yield: 50%; mp: 112–115 °C; ^1^H-NMR (400 MHz, DMSO) δ 9.82 (s, 1H, CONH), 7.85–7.67 (m, 4H, ArH), 7.27 (s, 2H, SO_2_NH_2_), 4.15 (q, *J* = 7.1 Hz, 2H, CH_2_), 1.93–1.79 (m, 4H, CH_2_), 1.18 (m, 4H, CH_2_), 1.17 (t, *J* = 7.1 Hz, 3H, CH_2_CH_3_), 0.89 (t, *J* = 7.2 Hz, 6H, Me). ^13^C-NMR (101 MHz, MeOD) δ 175.2, 172.4, 142.8, 140.2, 128.2, 121.6, 121.5, 62.7, 60.1, 38.6, 19.2, 14.7, 14.5. HRMS-ESI Calcd. for C_17_H_27_N_2_O_5_S [M + H]^+^: 371.1641; Found: 371.1610.

#### 3.2.5. 2-Propyl-2-(4-sulfamoylphenylcarbamoyl)pentanoic Acid **17**

*2-Propyl-2-(4-sulfamoylphenylcarbamoyl)pentanoic acid* (**17**): Yield: 50%; mp: 78–80 °C; ^1^H-NMR (400 MHz, DMSO) δ 9.96 (s, 1H, CONH), 7.80 (d, *J* = 9.0 Hz, 2H, ArH), 7.75 (d, *J* = 9.0 Hz, 2H, ArH), 7.27 (s, 2H, SO_2_NH_2_), 1.96–1.75 (m, 4H, CH_2_), 1.29–1.05 (m, 4H, CH_2_), 0.88 (t, *J* = 7.2 Hz, 6H, Me). ^13^C-NMR (101 MHz, MeOD) δ 177.9, 173.1, 142.6, 140.2, 128.3, 121.4, 59.7, 40.0, 19.5, 14.6. HRMS-ESI Calcd. for C_15_H_23_N_2_O_5_S [M + H]^+^: 343.1322; Found: 343.1296.

#### 3.2.6. General Produce for the Synthesis of Compounds **18a**–**c**

The synthesis of compounds **18a**–**c** was similar to the synthesis of compounds **12a**–**n**.

*N^1^-Allyl-2,2-dipropyl-N^3^-(4-sulfamoylphenyl)malonamide* (**18a**): Yield: 60%; mp: 189–192 °C; ^1^H-NMR (400 MHz, DMSO) δ 10.86 (s, 1H, CONH), 8.32 (t, *J* = 5.7 Hz, 1H, NH), 7.85–7.77 (m, 2H, ArH), 7.77–7.73 (m, 2H, ArH), 7.27 (s, 2H, SO_2_NH_2_), 5.90–5.72 (m, 1H, CH), 5.19–4.99 (m, 2H, CH_2_), 3.78 (t, *J* = 5.3 Hz, 2H, CH=CH2), 1.98–1.79 (m, 4H, CH_2_), 1.18–1.04 (m, 4H, CH_2_), 0.85 (t, *J* = 7.3 Hz, 6H, Me). ^13^C-NMR (101 MHz, MeOD) δ 175.9, 174.2, 142.8, 140.1, 128.2, 121.3, 58.9, 40.9, 40.4, 32.5, 21.2, 19.5, 14.6, 14.2. MS-ESI [M + H]^+^: 382.2. HRMS-ESI Calcd. for C_18_H_28_N_3_O_4_S [M + H]^+^: 382.1801; Found:382.1769.

*2,2-Dipropyl-N^1^-(4-sulfamoylphenyl)malonamide* (**18b**): Yield: 60%; mp: 202–205 °C; ^1^H-NMR (400 MHz, DMSO) δ 11.15 (s, 1H, CONH), 7.85–7.70 (m, 4H, ArH), 7.65 (s, 1H, NH), 7.50 (s, 1H, NH), 7.27 (s, 2H, SO_2_NH_2_), 1.93–1.65 (m, 4H, CH_2_), 1.21–1.03 (m, 4H, CH_2_), 0.84 (m, 6H, Me). ^13^C-NMR (101 MHz, MeOD) δ 179.2, 173.9, 142.8, 140.1, 128.2, 121.3, 59.0, 41.0, 19.5, 14.6. MS-ESI [M + H]^+^: 342.1. HRMS-ESI Calcd. for C_15_H_24_N_3_O_4_S [M + H]^+^: 342.1488; Found: 342.1460.

*N1,2,2-Tripropyl-N^3^-(4-sulfamoylphenyl)malonamide* (**18c**): Yield: 55%; mp: 189–192 °C; ^1^H-NMR (400 MHz, DMSO) δ 11.14 (s, 1H, CONH), 8.18 (t, *J* = 5.5 Hz, 1H, NH), 7.95–7.71 (m, 4H, ArH), 7.32 (s, 2H, SO_2_NH_2_), 3.20 (m, 2H, CH_2_), 2.02–1.80 (m, 4H), 1.55–1.39 (m, 2H, CH_2_), 1.31 (m, 4H, CH_2_), 1.21–1.08 (m, 4H, CH_2_), 0.91 (m, 9H, Me). ^13^C-NMR (101 MHz, MeOD) δ 175.9, 174.1, 142.8, 140.1, 135.5, 128.2, 121.4, 116.4, 59.1, 42.9, 40.7, 19.5, 14.6. MS-ESI [M + H]^+^: 384.2. HRMS-ESI Calcd. for C_18_H_28_N_3_O_4_S [M + H]^+^: 382.1801.; Found: 382.1786.

### 3.3. Pharmacology

#### 3.3.1. Preparation of the Compounds for Testing

All the tested compounds were suspended in 5% carboxymethyl cellulose sodium in sterilizedphysiological saline solution. The pentylenetetrazol was prepared by dissolution of pentylenetetrazol in sterilized physiological saline to make a 0.8% solution.

#### 3.3.2. MES Test

Kunming mice (18–22 g) purchased from Wuhan University Laboratory Animal Center were used in the MES test. After 0.5 h and 4 h of intraperitoneal (i.p.) administration of drugs at doses of 300 mg/kg, 100 mg/kg and 30 mg/kg, the mice were stimulated by an electrical stimulus (50 mA, 60 Hz, 0.2 s) through ear electrodes. Because the aim in this work was to find more effective and safer anticonvulsant drugs, the high, middle and low doses of drugs were chosen according to the effective dose of the drugs used clinically and reported previously by our group [21,22,24,25]. The procedure may cause the mice immediate hindlimb tonic extension. Protection against seizure was defined as the absence of hindlimb tonic extension. The duration of tonic seizures was analyzed over 5 min [35].

#### 3.3.3. scPTZ Test

After 0.5 h and 4 h of i.p. administration of drugs, the pentylenetetrazol solution was injected subcutaneously. Protection against pentylenetetrazol induced seizure was defined as the abolition of a threshold. All animals were observed for 30 min [36].

#### 3.3.4. Neurotoxicity Screening

The neurotoxicity of compounds was measured according to standardized rotorod test. Trained mice were placed on an accelerating rotarod (diameter 3.2 cm) rotating at 10 rpm after drug administration. Neurotoxicity was defined as the inability of the mice to keep balance on the rotarod fpr at least 1 min

#### 3.3.5. Calculation of ClogP

ClogP was calculated by using ChemDraw-Ultra software (Version 6.0.1; PerkinElmer Informatics, Waltham, MA, USA).

#### 3.3.6. Quntification Studies

For the determination of the median effective dose (ED_50_), groups of 10 mice were given a range of i.p. doses (160 mg/kg, 80 mg/kg, 40 mg/kg, 20 mg/kg, and 10 mg/kg) of the tested compounds. Similarly, for the determination of median toxic dose (TD_50_), mice were given a range of i.p. doses (100 mg/kg, 300 mg/kg, 500 mg/kg, 700 mg/kg, and 900 mg/kg) of the tested compounds. These data were subjected to Graphpad prism 5 to calculate the ED_50_, TD_50_ and the 95% confidence interval.

## 4. Conclusions

A novel series of benzenesulfonamide derivatives containing 4-aminobenzenesulfonamide and α-amides branched valproic acid or 2,2-dimethylcyclopropanecarboxylic acid moieties were synthesized in good yields and screened for their anticonvulsant activities in MES and scPTZ test. Compounds **12c**, **12d** and **18b** showed outstanding anticonvulsant activities in the MES test or scPTZ test. The highest activity in the MES test was observed for compound **18b** with an ED_50_ value of 16.36 mg/kg. The most active compound in the scPTZ test was compound **12c**, which was seven times more active than that of valproate. Meanwhile, these three compounds have lower toxicity compared to phenytoin. The least toxic compound was compound **12d**, with a LD_50_ value of 992.0 mg/kg, which was nine times more than that of phenytoin. All three of these compounds exhibited better protective index than phenytoin and valproate, which indicated they can be used as lead compounds for future investigation to discover more effective and safer anticonvulsant drugs.

## Supplementary Materials

Supplementary materials can be accessed at: http://www.mdpi.com/1420-3049/20/09/17585/s1.
