# Supplementary material for: Synthesis and Pharmacological Evaluation of Novel Benzenesulfonamide Derivatives as Potential Anticonvulsant Agents
Source: Molecules. 2015 Sep 23;20(9):17585–600. doi: 10.3390/molecules200917585 (PMC6331867; doi:10.3390/molecules200917585)
Supplement: Supplementary file 1 [file molecules-20-17585-s001.pdf]

Supplementary Materials

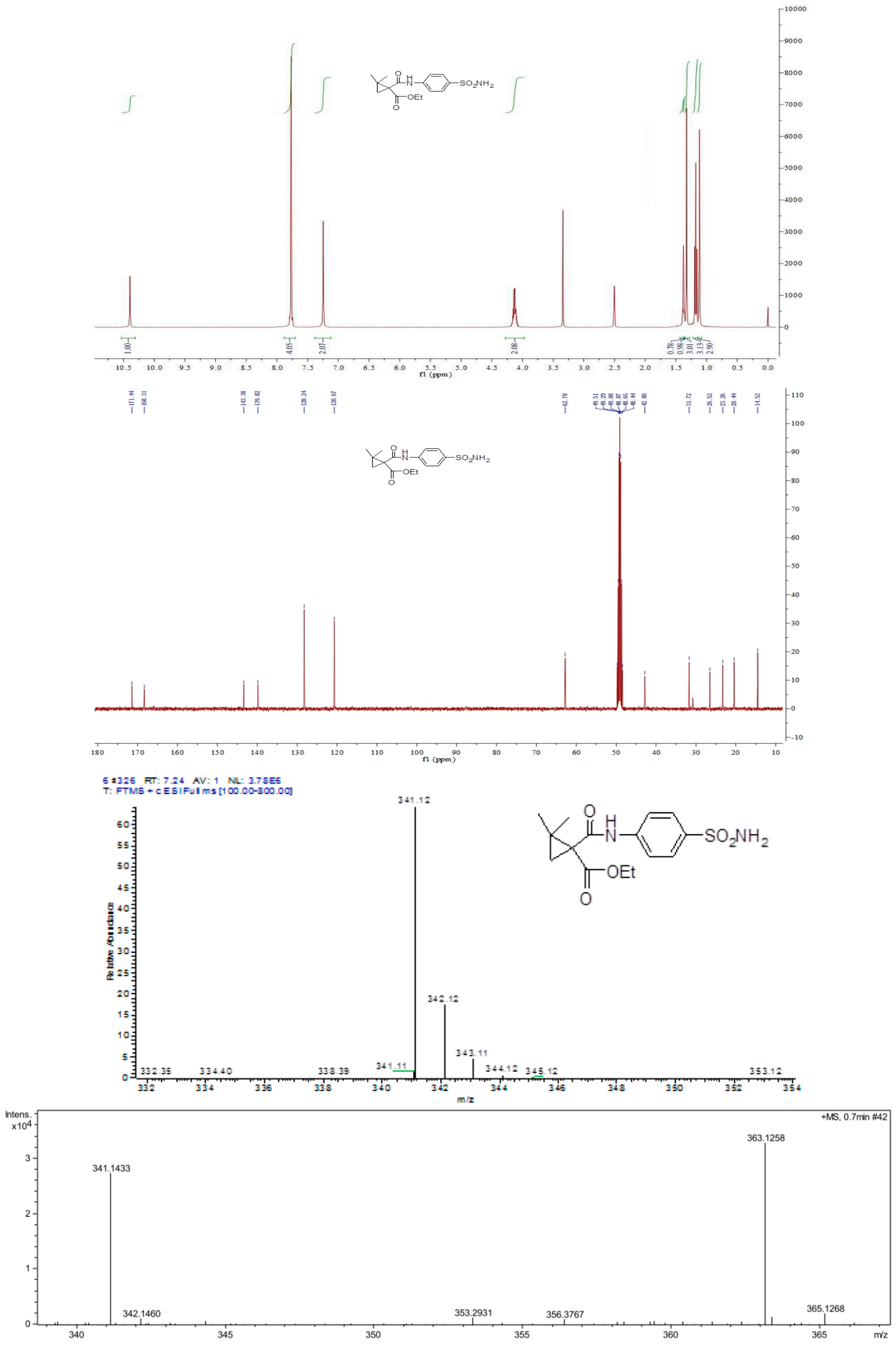

Figure S1. ^{1}H-NMR, ^{13}C-NMR, MS-ESI and HRMS spectrums of compound **10**.

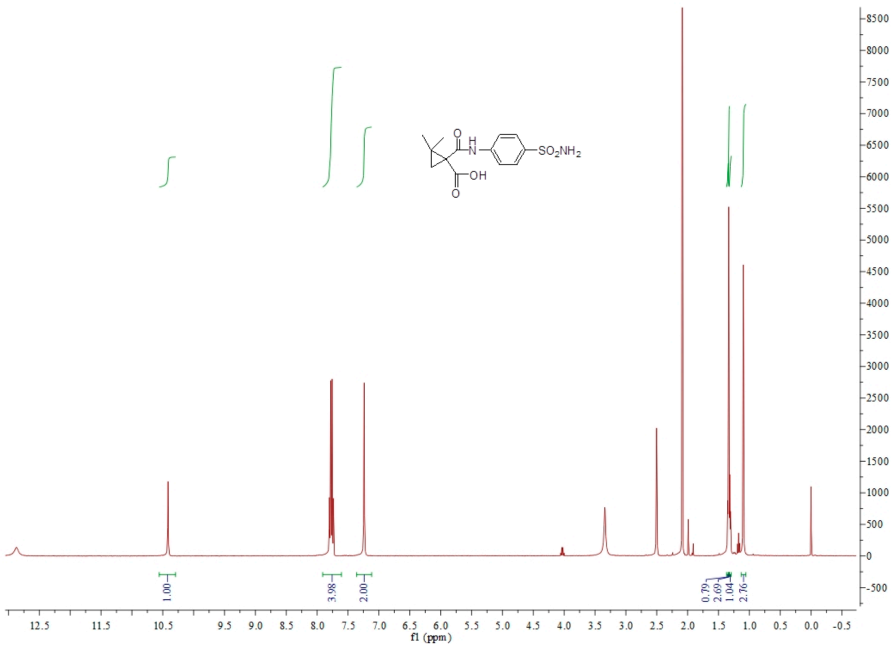

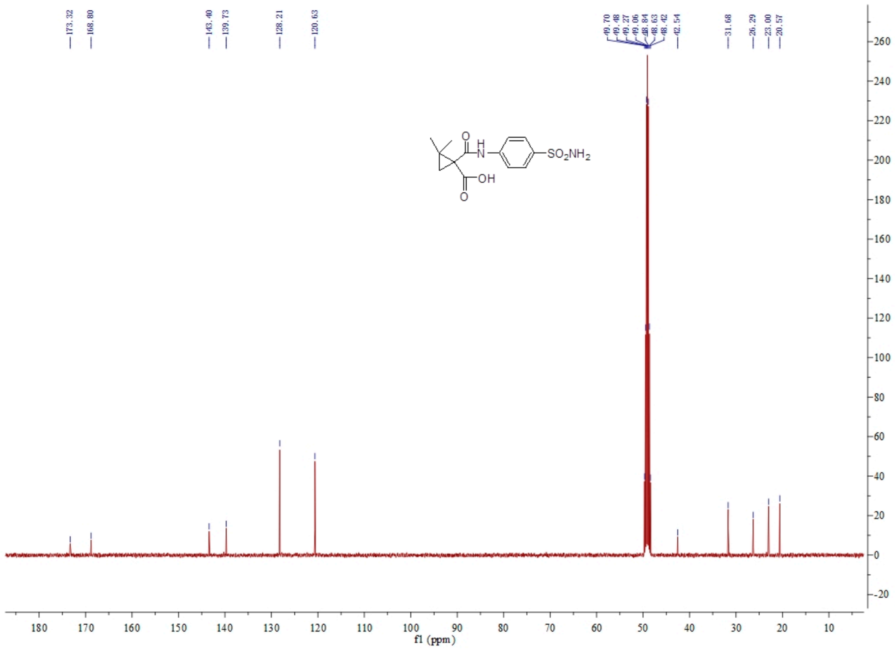

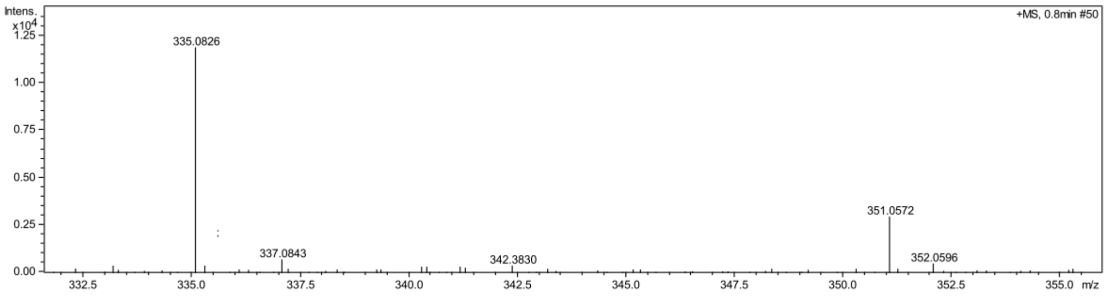

Figure S2. ^{1}H-NMR, ^{13}C-NMR and HRMS spectrums of compound **11**.

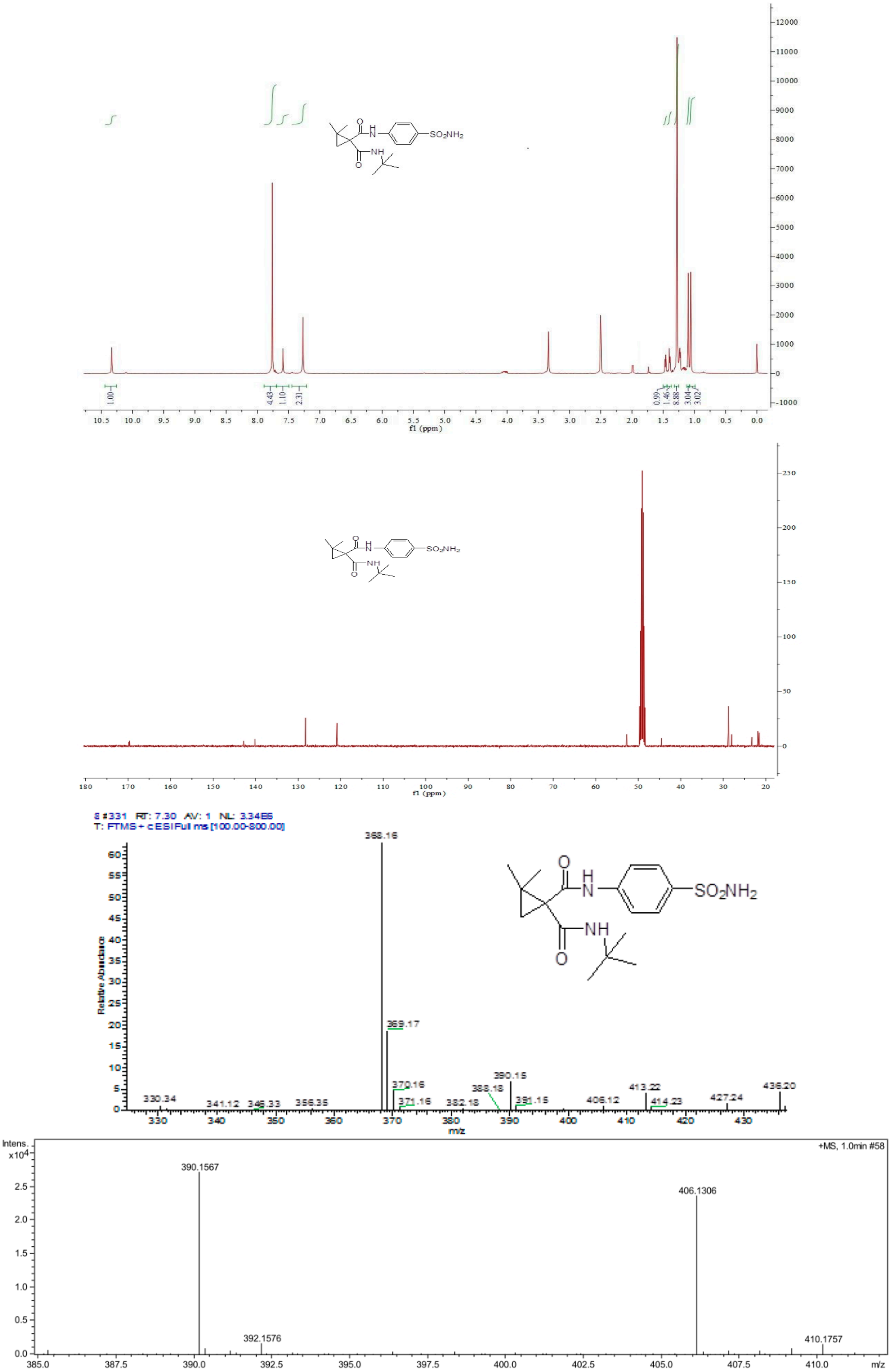

Figure S3. ^{1}H-NMR, ^{13}C-NMR, MS-ESI and HRMS spectrums of compound **12a**.

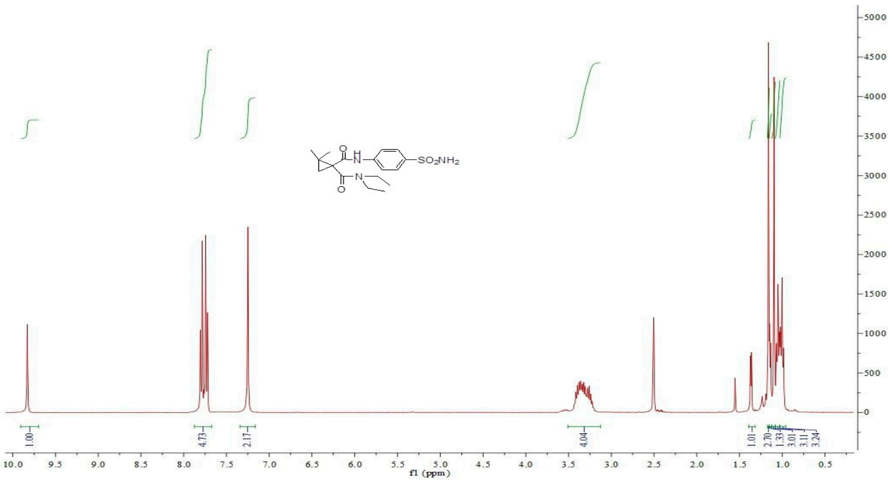

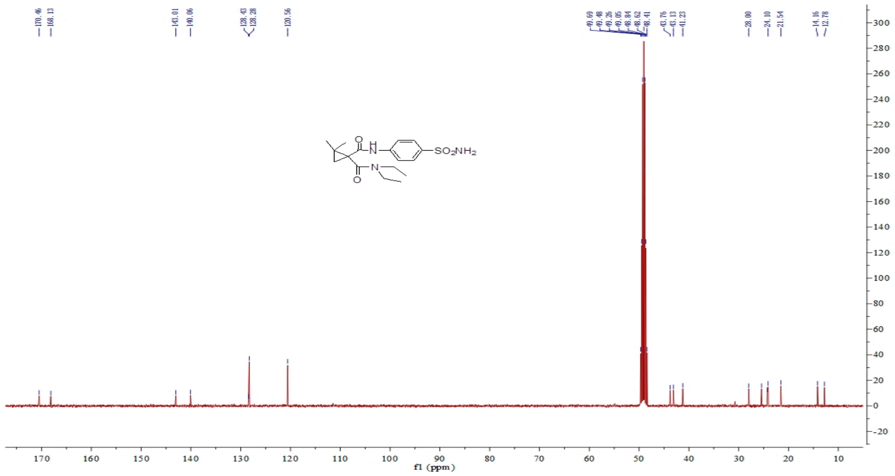

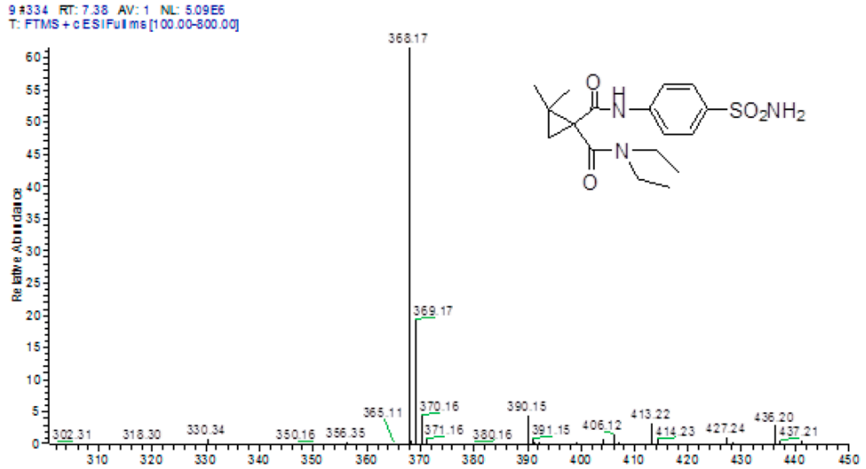

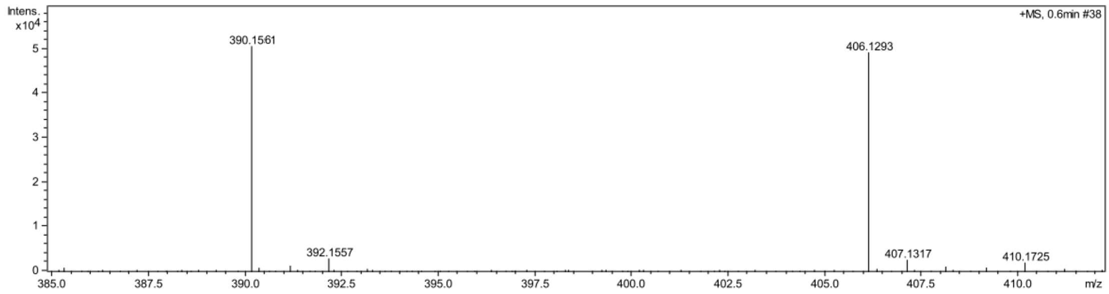

Figure S4. ^{1}H-NMR, ^{13}C-NMR, MS-ESI and HRMS spectrums of compound **12b**.

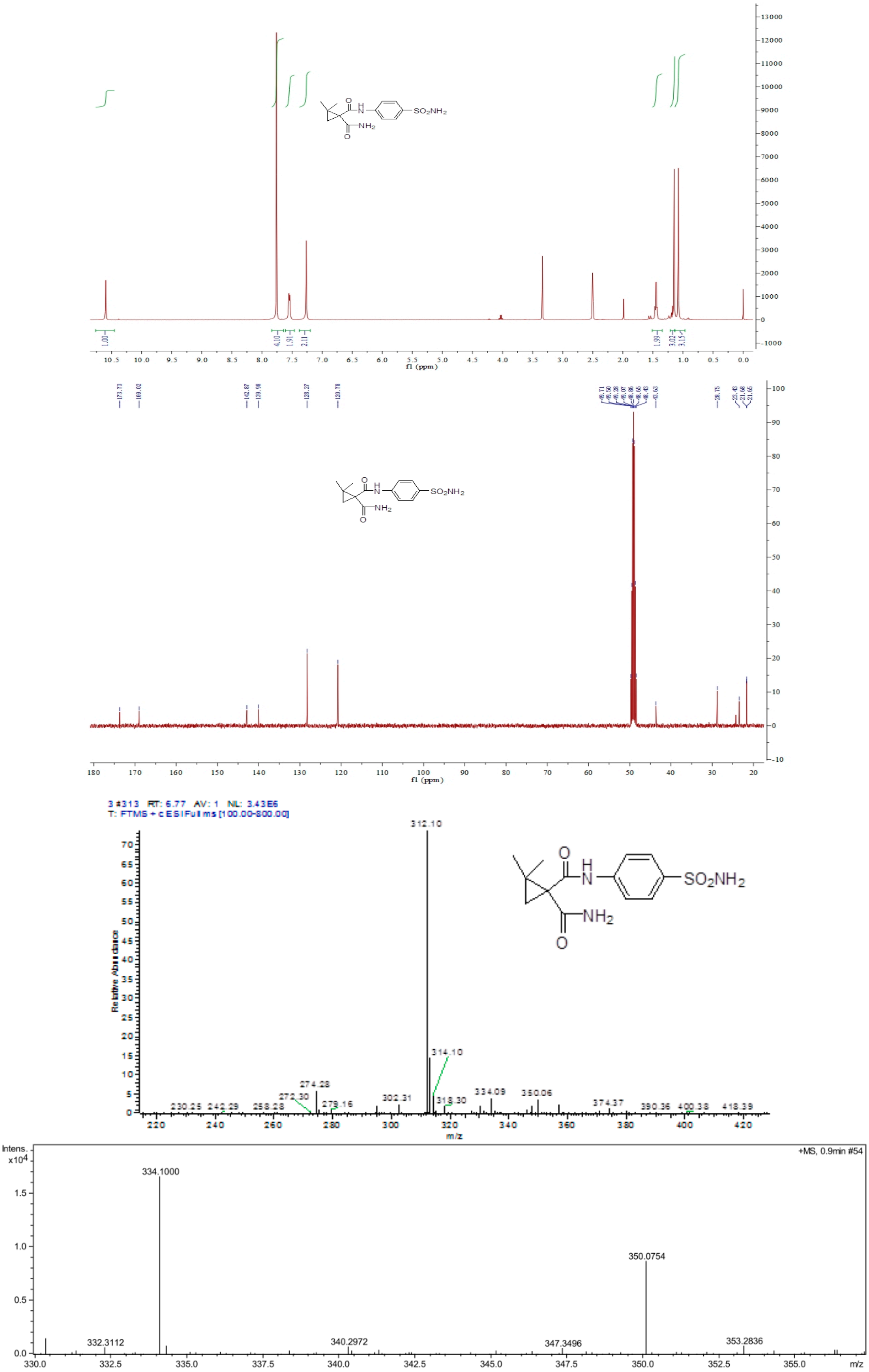

Figure S5. ^{1}H-NMR, ^{13}C-NMR, MS-ESI and HRMS spectrums of compound **12c**.

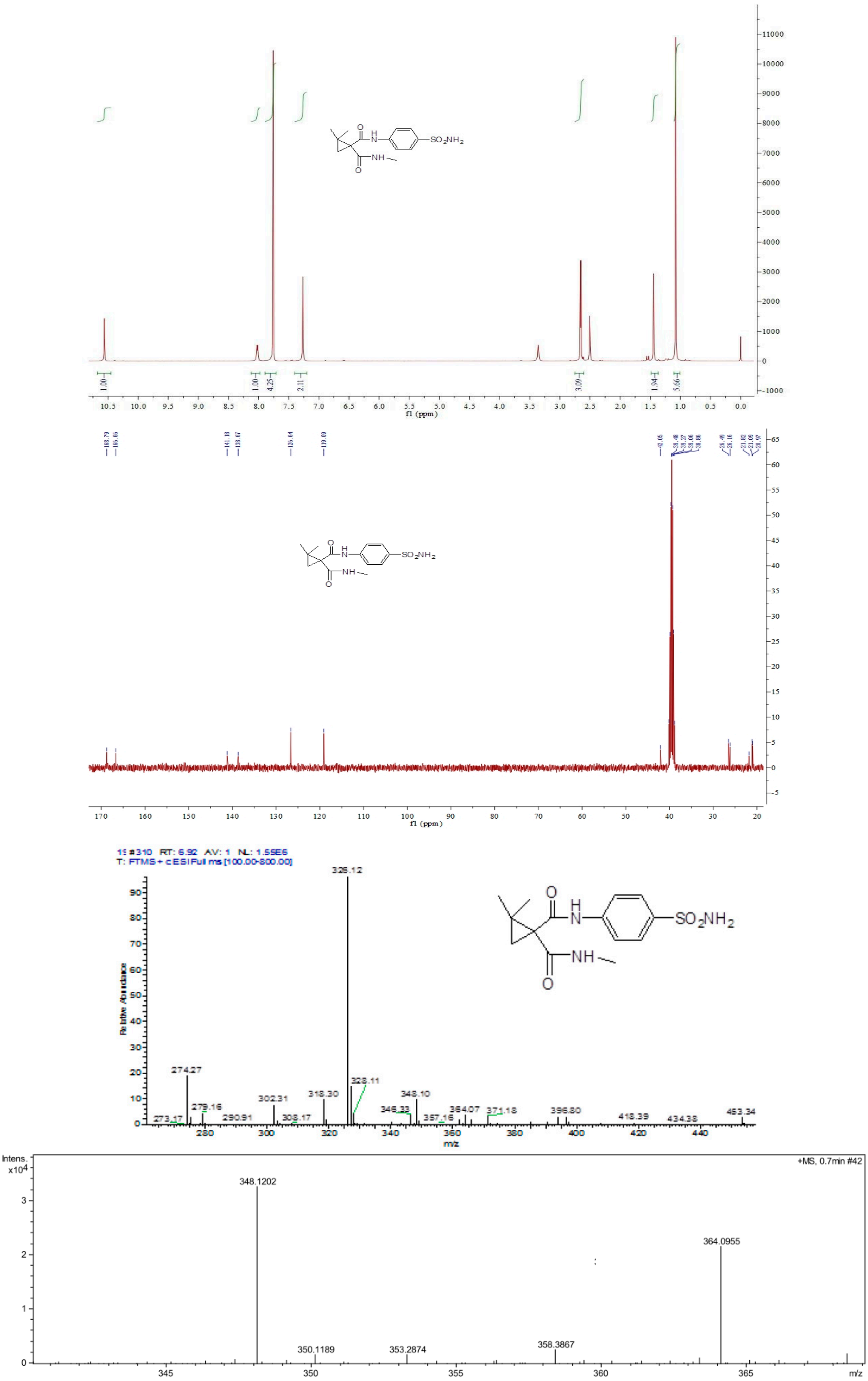

Figure S6. ^{1}H-NMR, ^{13}C-NMR, MS-ESI and HRMS spectrums of compound **12d**.

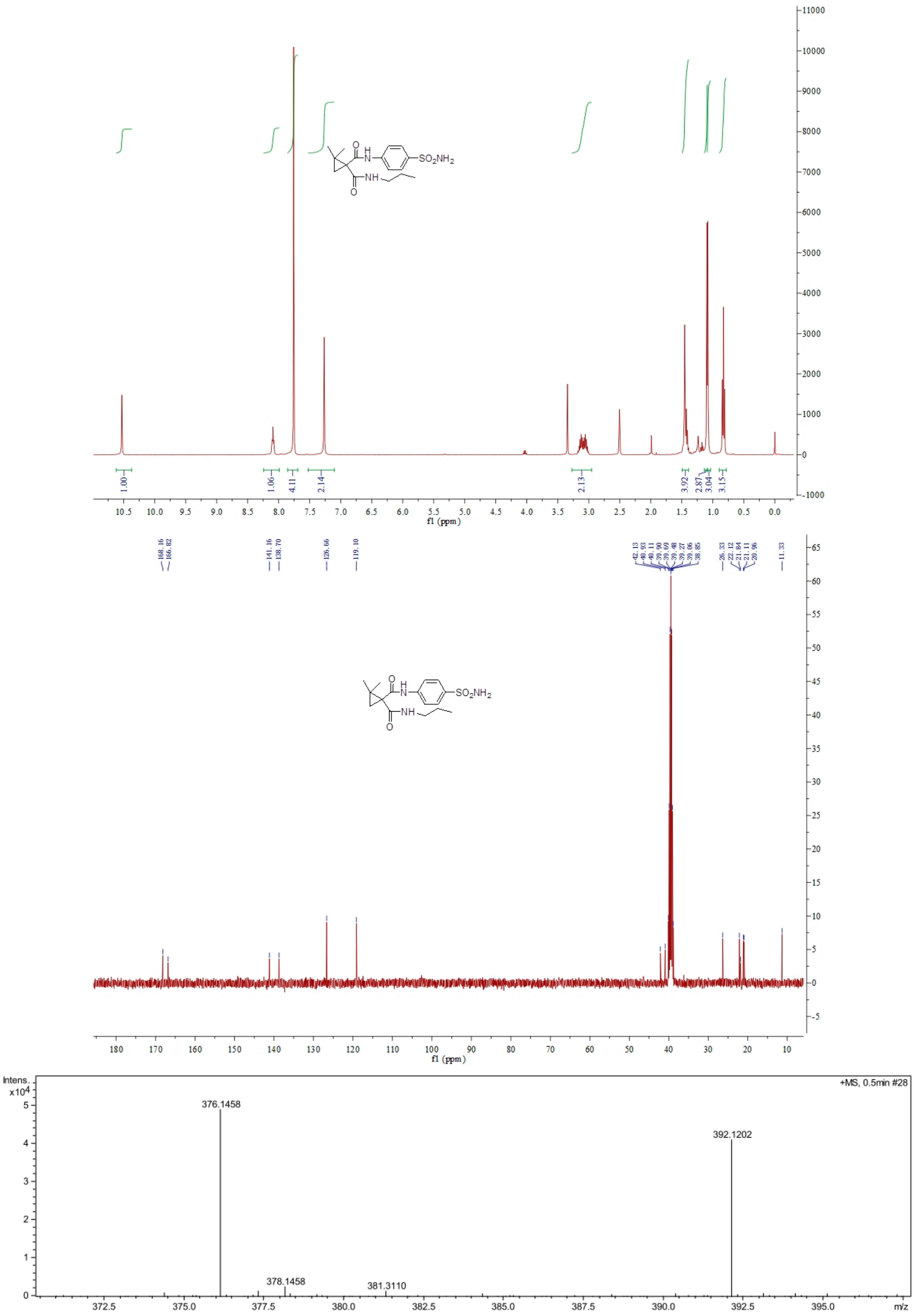

Figure S7. ^{1}H-NMR, ^{13}C-NMR and HRMS spectrums of compound **12e**.

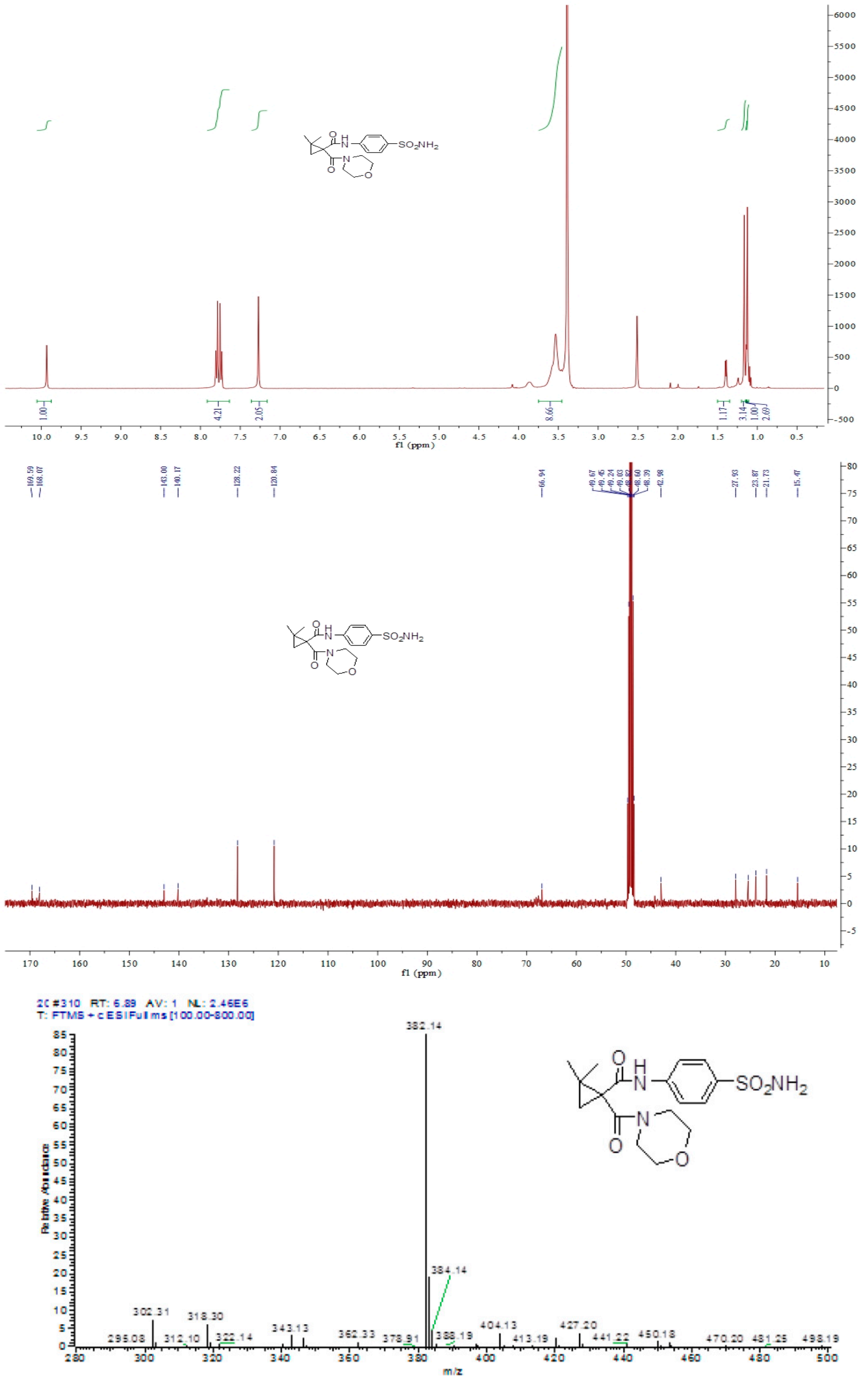

Figure S8. ^{1}H-NMR, ^{13}C-NMR and MS-ESI spectrums of compound **12g.**

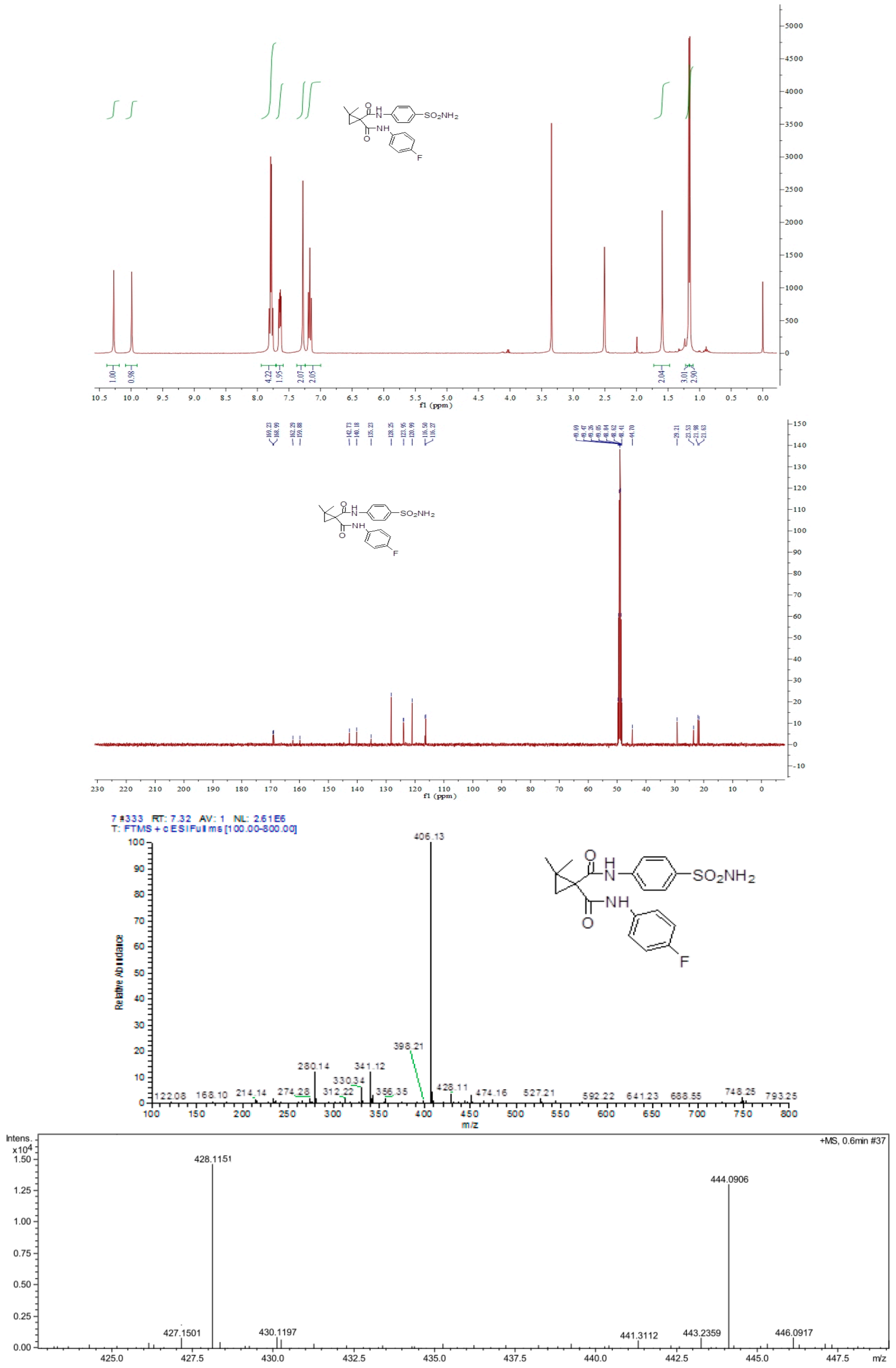

Figure S9. ^{1}H-NMR, ^{13}C-NMR, MS-ESI and HRMS spectrums of compound **12h**.

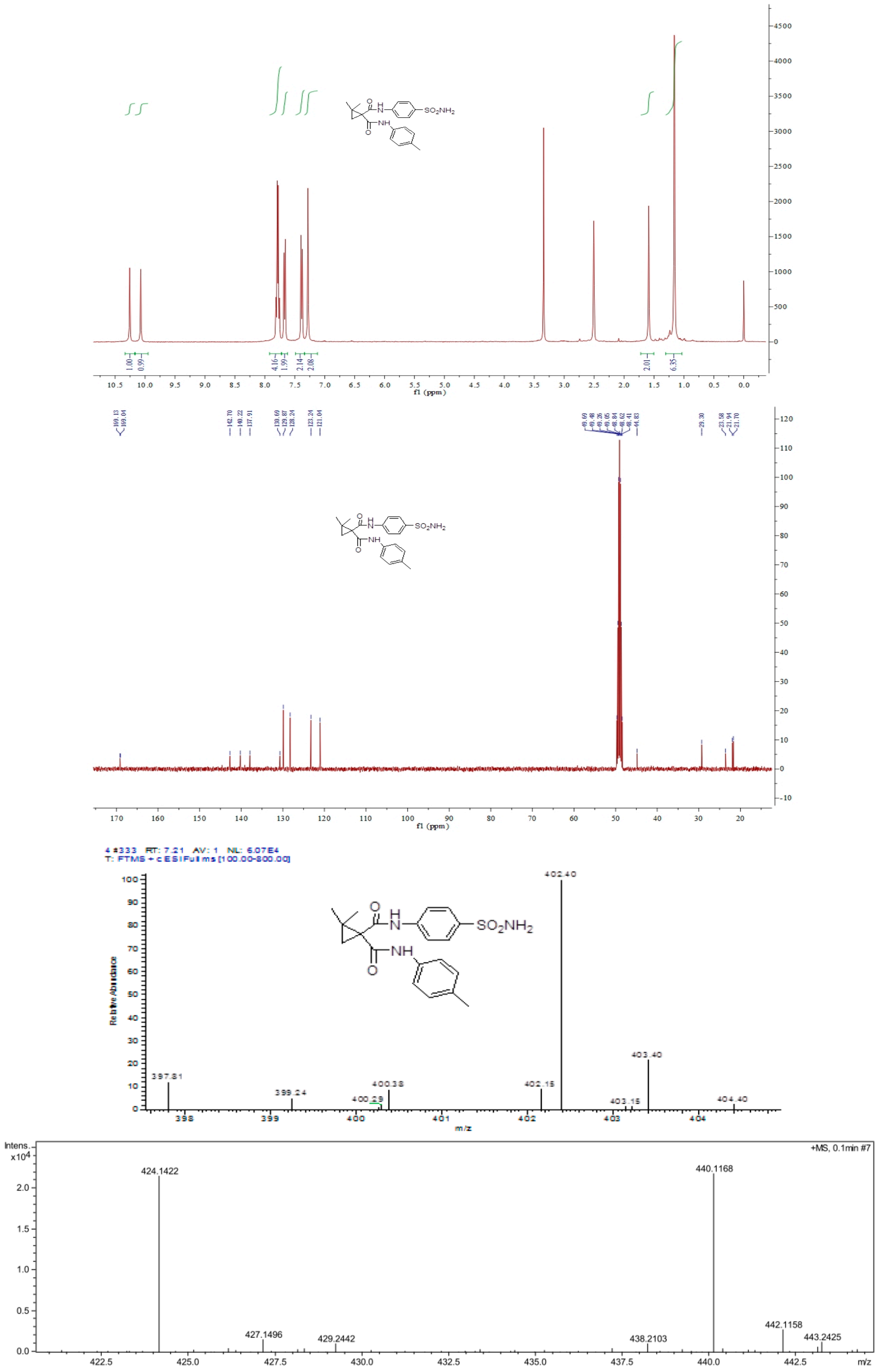

Figure S10. ^{1}H-NMR, ^{13}C-NMR, MS-ESI and HRMS spectrums of compound **12i**.

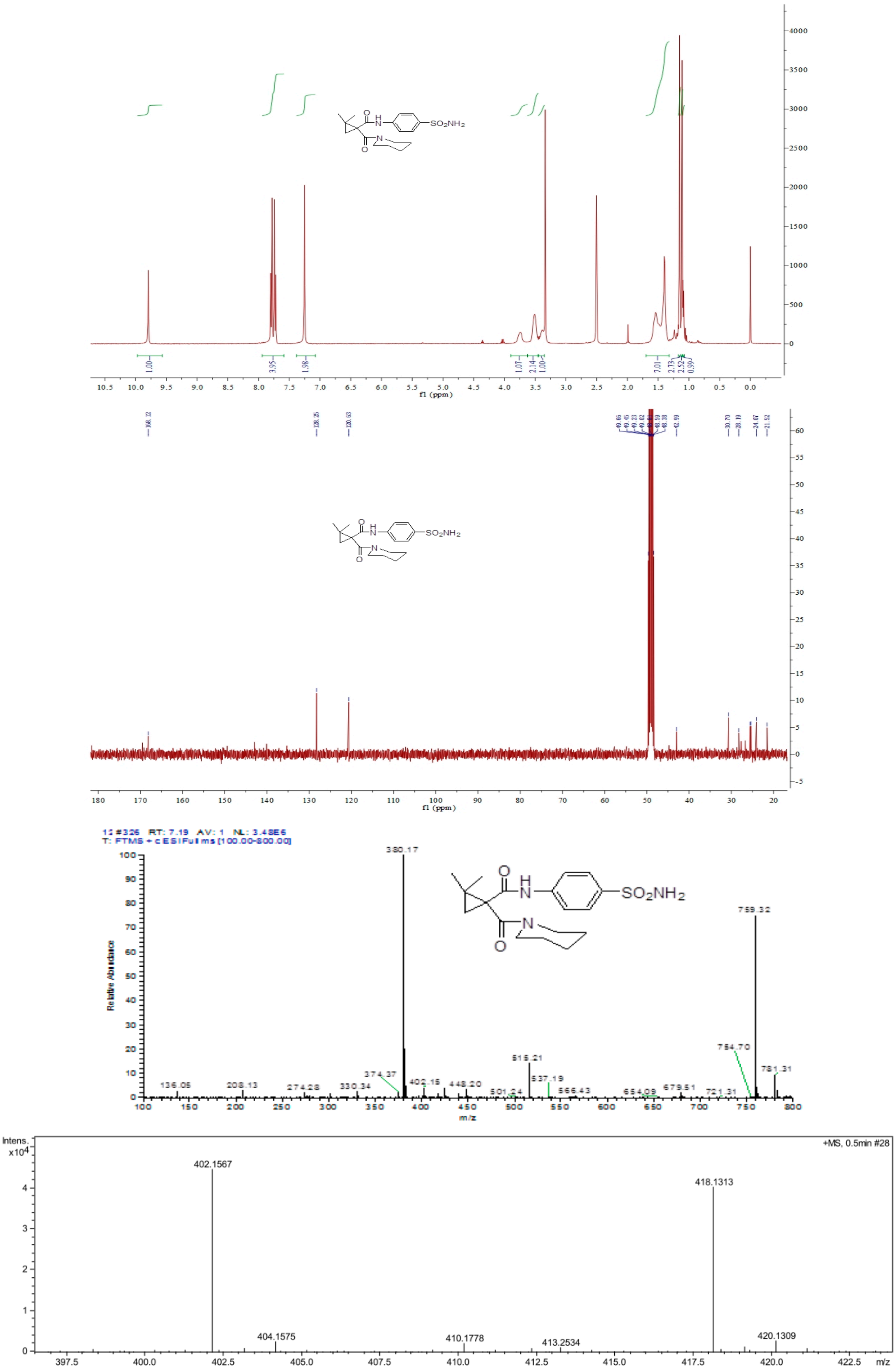

Figure S11. ^{1}H-NMR, ^{13}C-NMR, MS-ESI and HRMS spectrums of compound **12j**.

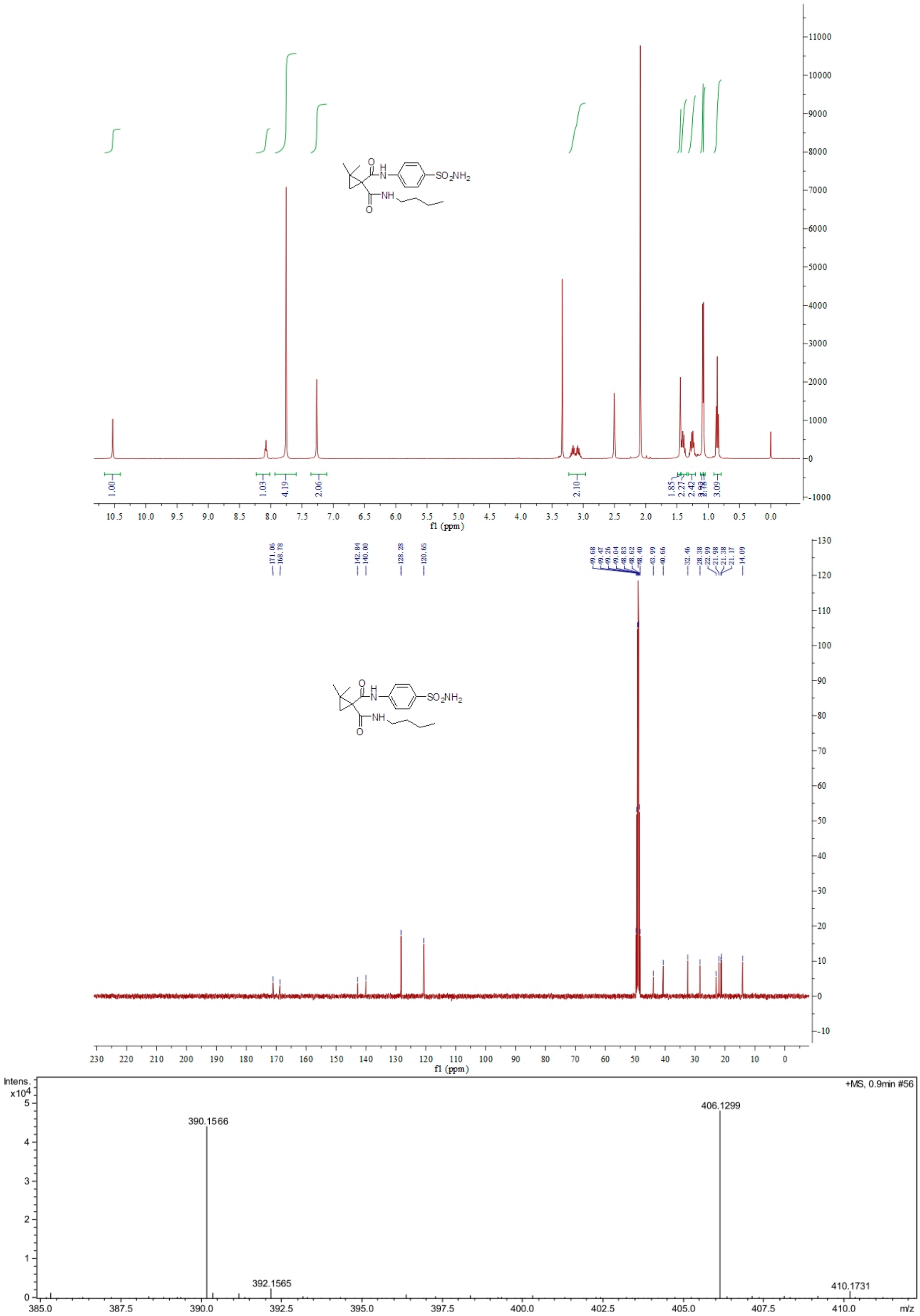

Figure S12. ^{1}H-NMR, ^{13}C-NMR and HRMS spectrums of compound **12k**.

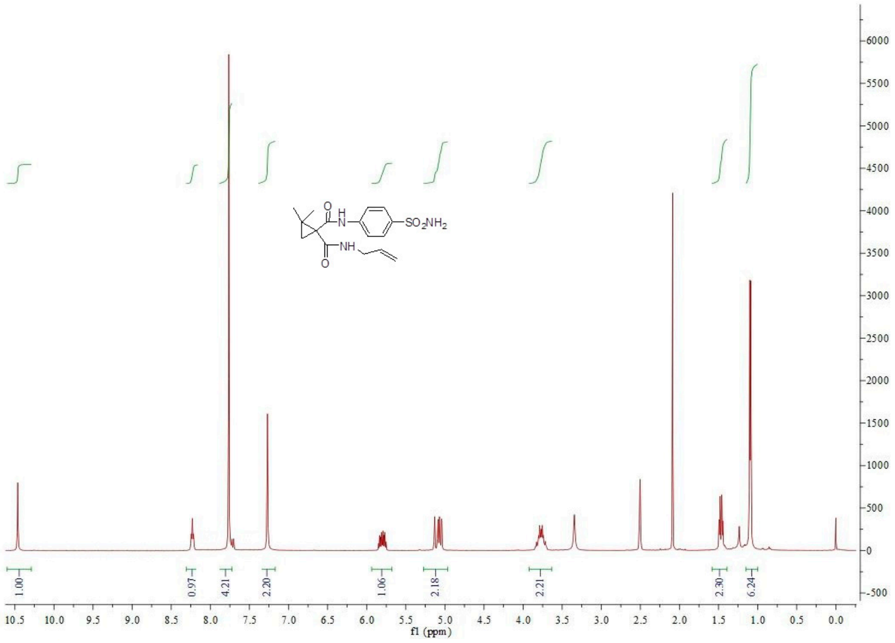

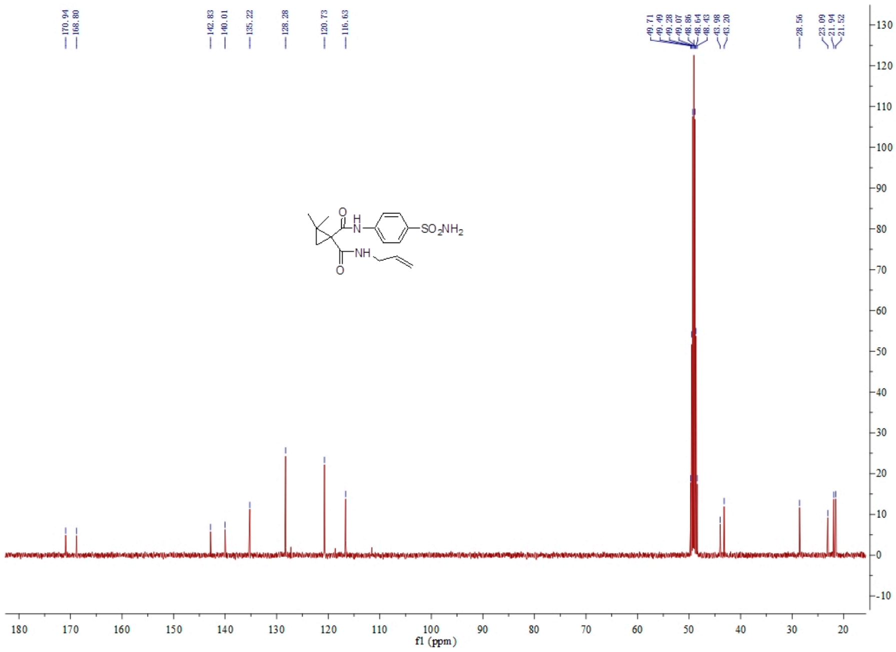

Figure S13. ^{1}H-NMR and ^{13}C-NMR spectrums of compound **12n**.

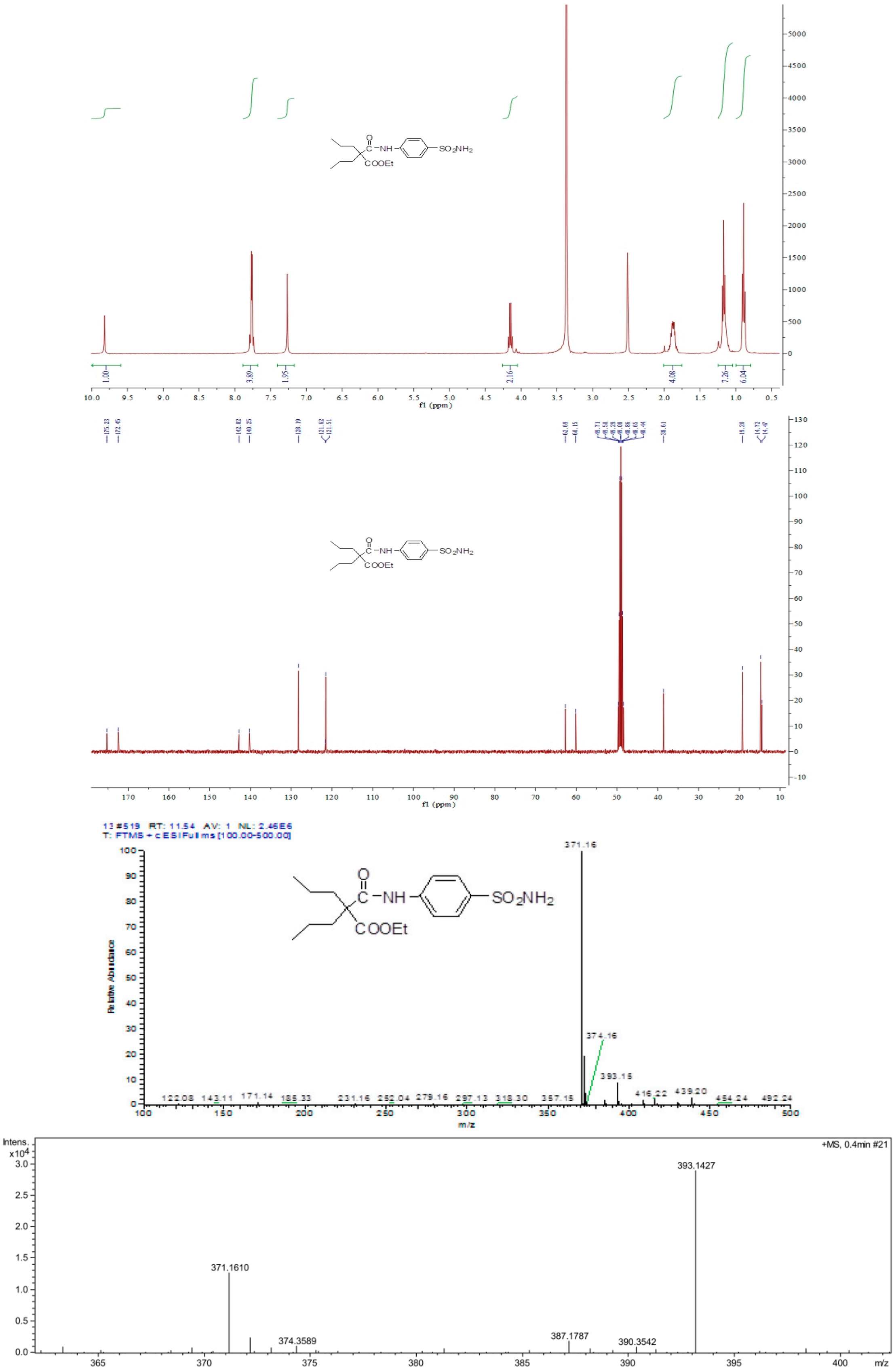

Figure S14. ^{1}H-NMR, ^{13}C-NMR, MS-ESI and HRMS spectrums of compound **16**.

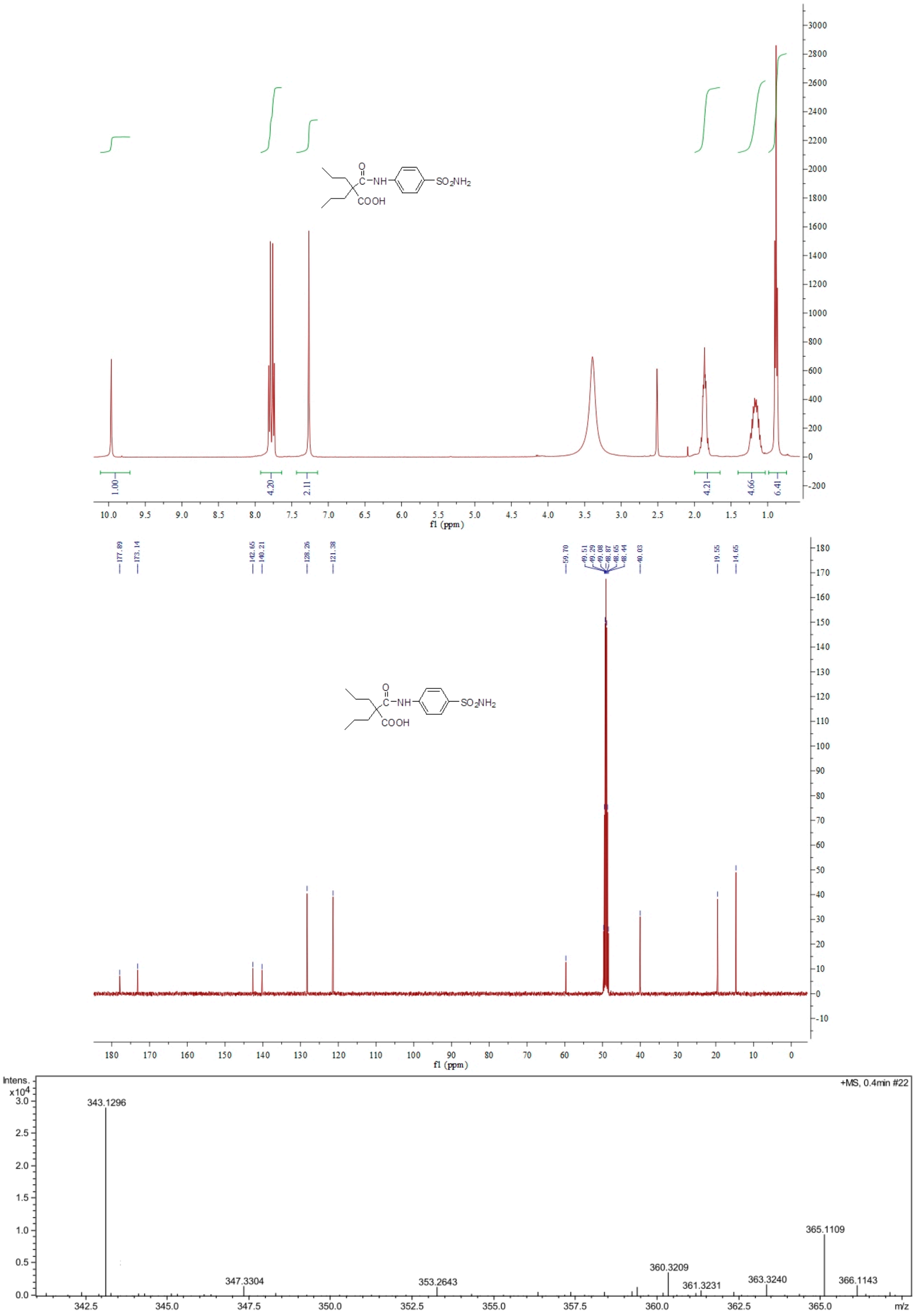

Figure S15. ^{1}H-NMR, ^{13}C-NMR and HRMS spectrums of compound **17**.

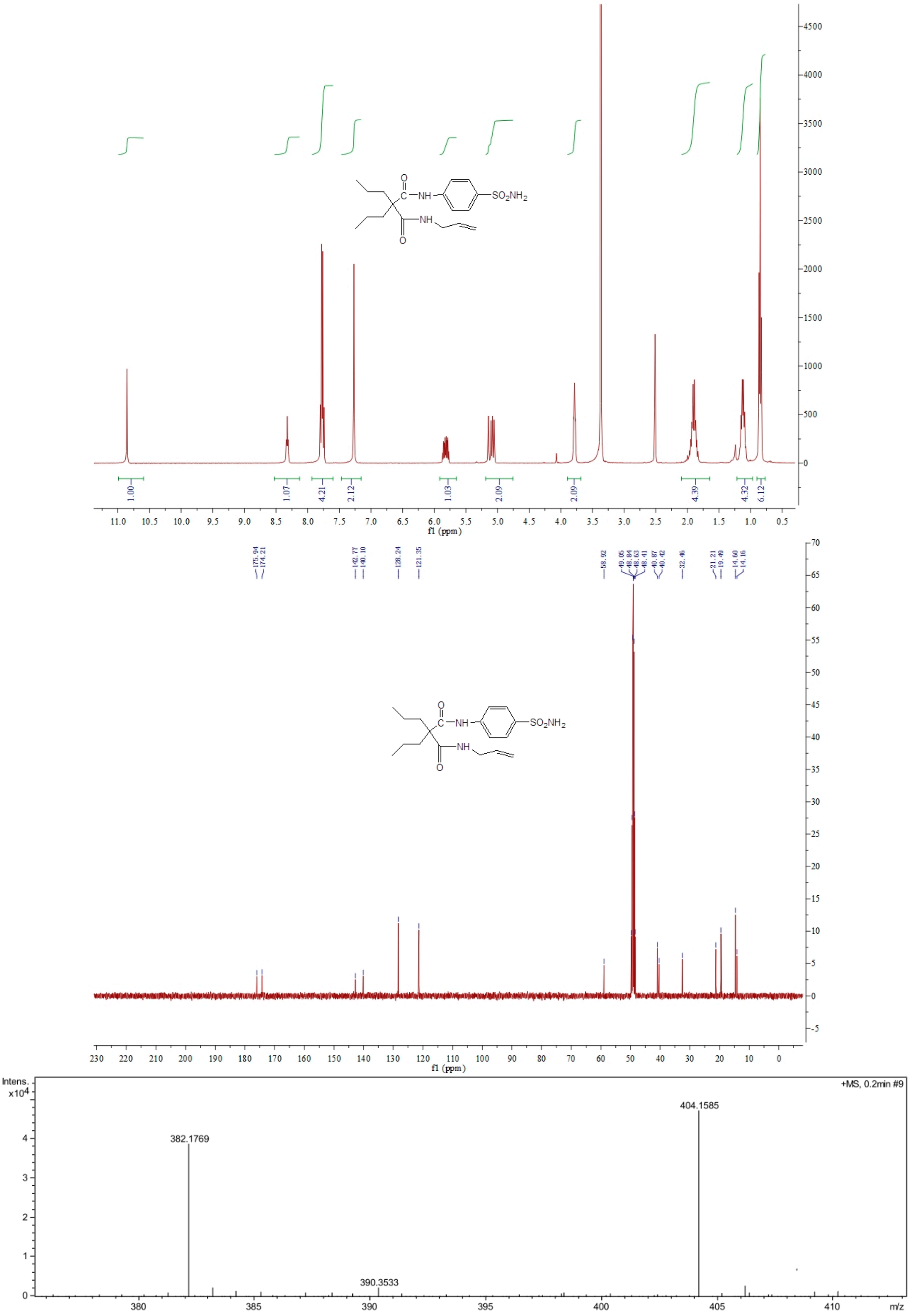

Figure S16. ^{1}H-NMR, ^{13}C-NMR and HRMS spectrums of compound **18a**.

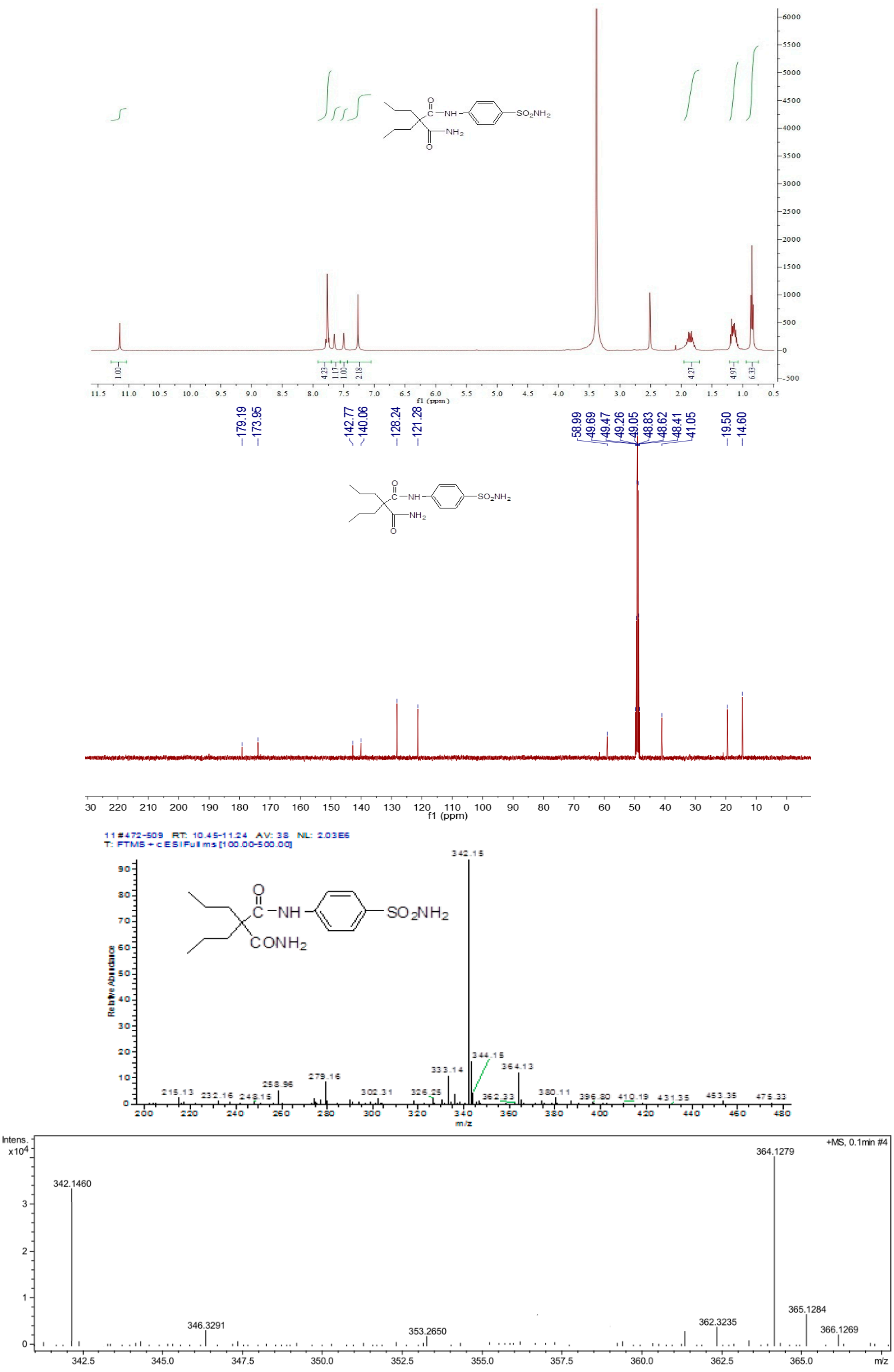

Figure S17. ^{1}H-NMR, ^{13}C-NMR, MS-ESI and HRMS spectrums of compound **18b**.

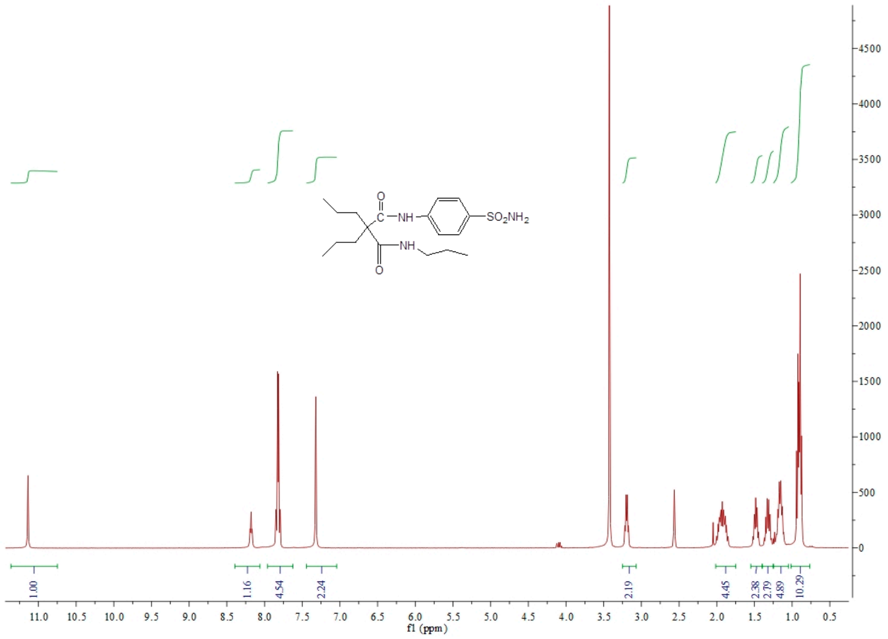

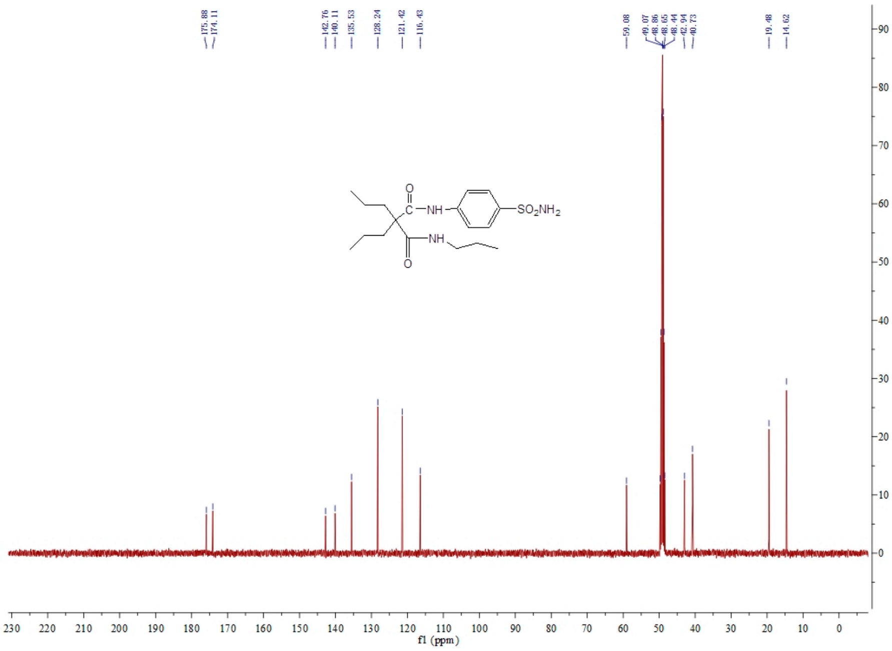

Figure S18. ^{1}H-NMR and ^{13}C-NMR spectrums of compound **18c**.
